# Helping Trainees Develop Scholarship in Academic Medicine From Community Service

**DOI:** 10.15766/mep_2374-8265.10659

**Published:** 2017-12-14

**Authors:** Sunny Nakae, Maria Soto-Greene, Renee Williams, Daniel Guzman, John P. Sánchez

**Affiliations:** 1 Assistant Dean of Admissions, Recruitment & Student Life, Loyola University Chicago Stritch School of Medicine; 2 Vice Dean, Rutgers New Jersey Medical School; 3 Professor of Medicine, Rutgers New Jersey Medical School; 4 Assistant Professor of Medicine, Division of Gastroenterology, New York University School of Medicine; 5 Fourth-Year Medical Student, Rutgers New Jersey Medical School; 6 Assistant Dean for Diversity and Inclusion, Rutgers New Jersey Medical School; 7 Associate Professor, Rutgers New Jersey Medical School

**Keywords:** Scholarship, Academic Medicine, Community Service

## Abstract

**Introduction:**

Service in the community and academic medicine are often seen by trainees as unrelated. This may be one reason for the lack of faculty diversity and the declining interest in academic medicine among new trainees.

**Methods:**

We developed an educational workshop through the application of the Kern model to help medical students and residents understand the relationship between community service work and scholarship as it pertains to a career in academic medicine. Specifically, the workshop helped trainees (1) understand the terms service and scholarship, (2) understand the benefits of achieving community service scholarship, and (3) identify steps to achieve community service scholarship through mock cases and personal stories.

**Results:**

The workshop was implemented at five conferences with a total of 139 trainees. Results of a paired-samples t test of learners' responses pre- and postworkshop showed statistically significant growth in their confidence to publish service-related work, as well as more positive agreement with the notion that community service work aligns with an academic medicine career.

**Discussion:**

This effective module can help trainees understand how community service and academic medicine are aligned, and raise their confidence in building a foundation for an academic medicine career through conducting community service scholarship.

## Educational Objectives

At the completion of this module, learners will be able to:

1. Define the terms *community service, community engagement,* and *service learning.*
2. Describe frameworks to help achieve community service scholarship.
3. Apply the following steps to achieving community service scholarship: sample cases and personal stories.

## Introduction

Academic career development is key for ensuring talent in academic medicine in the long term. Often, students perceive academic medicine and community service as divergent career paths, despite community service being a core tenet of academic medicine^1^ and a component of Liaison Committee on Medical Education accreditation standards.^2^ The perception of academic careers as narrow and oriented to bench research can serve as a barrier to trainees considering and pursuing academic medicine.^3,4^ This barrier can be especially limiting for underrepresented minorities and sexual minorities, who may have higher propensities to provide service in underserved communities throughout their careers.^3,5^

According to the 2016 AAMC Medical School Graduation Questionnaire, 83.6% and 52.2% of graduates expect to engage in teaching and research activities, respectively, during their careers; however, only 45.3% and 28.6% expect to serve as faculty and administration, respectively, during their careers.^6^ Jeffe, Yan, and Andriole found that racial and ethnic minority medical students were less likely than their white counterparts to report an interest in pursuing academia on medical school entry and were more likely to report diminished intent on graduation.^7^ Contributing to disinterest in medical students identified as underrepresented in medicine are such factors as the perception that community service is not valued in the promotion process, cultural taxation, and the challenge of balancing native cultural ties and values with the culture of academic medicine.^3,5^ Similarly, for lesbian, gay, bisexual, and transgender (LGBT) trainees, there is great interest in participating in LGBT-related health education, service, and research but also a fear of the impact of being out, personally or through their work, on their academic success.^8^ Medical schools must intervene earlier to introduce trainees to pathways toward academic medicine that include robust opportunities for community service scholarship. This will facilitate student pathways that assist in integrating various components of an academic career that may be more widely appealing, thereby increasing participation by all groups, including those underrepresented.

Engagement in service-related work, especially when structured as service learning, can facilitate trainees' intra-/interpersonal skills (teamwork and collaboration, leadership, communication, and diversity), academic and professional skills (clinical skills and subject-specific knowledge, problem analysis and critical thinking, self-confidence and efficacy, physician role and working environment/specialty, and understanding of public health determinants and policy), civic engagement and social responsibility (social justice and support for community), and career intentions.^9–13^ As service-related work is also considered on the American Medical College Application Service and the Electronic Residency Application Service applications, for Alpha Omega Alpha designation, and on faculty portfolios,^14^ it can greatly affect career trajectory along multiple steps of an individual's educational journey.^15^ The achievement of service-based scholarship, on top of service work, can facilitate and strengthen a path towards academia.

There is published instruction on developing, implementing, and evaluating service-related work. Much instruction on community service work describes how to incorporate teaching about cultural competency and health disparities and the relevance and process of service learning, collaborative community health research, and community-engaged participatory research.^16–20^ Not detailed is the relevance or method of transforming service-related work into scholarship for an academic medicine trajectory. The ongoing engagement of trainees in community service despite their perception of its divergence from academia, as well as academic medical centers' increasing need for diverse faculty and leaders, stresses the need for greater instruction on the relevance and methods of completing service scholarship.

As previously described, Building the Next Generation of Academic Physicians (BNGAP) developed a set of workshops (one titled “Introducing Trainees to Medical Education Activities and Opportunities for Educational Scholarship” published in *MedEdPORTAL*^21^) to heighten medical students' and residents' awareness of academic careers. A BNGAP curriculum committee comprising 25 diverse trainees and educational leaders from across the country helped to create and/or review the workshops. This current workshop focuses on describing service work, its relevance, and how to transform it into scholarship. Four coauthors (Sunny Nakae, Maria Soto-Greene, Renee Williams, and John P. Sánchez) of the workshop have experience in developing, evaluating, and disseminating service-related content. The six-step Kern model was applied by the curriculum committee members as a framework for the design, implementation, and evaluation of the workshops:^22^

1. *Problem identification and general needs assessment:* This step was performed via literature review and input from trainees and faculty.
2. *Targeted needs assessment:* The assessment was made via a mixed-methods study of trainees' perceptions of academic medicine careers, including facilitators and barriers to academic career intent, and preferred career development activities.^3,5,8,23–25^
3. *Goals and objectives:* Based on the literature review, results of the mixed-methods study, and committee member input, the goals of the workshop are to teach participants to define the terms *community service, community engagement,* and *service learning* and describe frameworks to help achieve community service scholarship. This is accomplished by applying the following learning methodologies: sample cases and personal stories.
4. *Educational strategies:* To stimulate an active learning environment, the material was presented via an interactive workshop that incorporated case-based small-group discussions. Small groups have a positive effect on learning performance by promoting learner motivation and authenticity, as well as active participation, purposeful activity, and face-to-face contact.^26,27^
5. *Implementation:* The 1.5-hour workshop was administered during an academic medicine career development conference for medical students and residents. Participants and speakers came from the hosting medical school and from nearby academic health centers. This venue was chosen because it afforded students opportunities for career-specific learning, skill development, positive learning environments, and networking with individuals beyond their own academic health center.
6. *Evaluation and feedback:* Each conference participant was asked to complete a questionnaire and evaluate the workshop design and content.

Social cognitive career theory (SCCT) is another strong theoretical foundation of the BNGAP workshops. SCCT posits that learners are more likely to pursue career areas where they perceive high self-efficacy. Self-efficacy beliefs are generated based on exposure, role modeling, mentoring, and observation. Therefore, students must have robust exposure to academic careers with clear understanding of pathways that align with their motivations in order to subsequently decide to pursue academic medicine. Our workshop aimed to heighten trainees' self-efficacy by reviewing familiar service-related case scenarios and discussing how their content could be published, and utilizing the testimony of faculty who share their experiences of completing service scholarship.

This workshop has been implemented in the context of a larger curriculum but can also be run as a stand-alone module. The workshop includes core concepts regarding service-related activities and how to transform work into scholarship that can be utilized across health professions (e.g., dental, nursing, and physician assistant). If the workshop is adapted, we recommend using speakers and cases that resonate with the respective audience. At a minimum, the hope is that workshop participants will experience transformative learning by questioning their assumptions and beliefs about service work and its alignment with a career in academic medicine.

## Methods

Two educational strategies are featured in this workshop: (1) an interactive didactic component, via PowerPoint (PPT) presentation, to introduce students to basic knowledge and concepts related to service work and its transformation into scholarship, and (2) small-group learning for participants to apply newly acquired knowledge in discussing mock cases of common student projects. The PPT presentation highlights the use of frameworks and models to guide the development, implementation, and evaluation of service projects and improve project designers' ability to complete service scholarship. Frameworks/models discussed in the PPT include community health research, the Kern model, the Cené model, and the SMART model.^19,22,28–29^ The cases are meant to provide personal context, highlight how to categorize service activities, and incorporate frameworks to achieve service scholarship (i.e., publication or presentation). Each session should be restricted to no more than 50 medical students and residents to create a safe space to discuss their personal perspectives, professional ambitions, and challenges in respect to their future medical education careers.

In preparation for this workshop, facilitators should review Appendices A–F. The flow and content of this workshop are featured in Appendix A's 33- slide PPT presentation. The presentation outlines the core content for participants, including key terms and definitions, concepts and best practices to consider in transforming service work into scholarship, and three case scenarios for participants to apply what was outlined in the preceding slides. Appendix B provides step-by-step instructions for conducting the workshop along with an explanation of how to discuss each slide in the PPT. Facilitators are encouraged to include their own personal experiences for authenticity. For example, slide 28 features service work by Dr. John P. Sánchez, coauthor of this workshop, on a service-related project he implemented in medical school and residency. This can be replaced by the facilitator's own experiences and publications. Appendix C is an adjunct to the discussion guide to help visual and audio learners gain an appreciation of how to implement the workshop. This 16.5-minute video features Dr. Sánchez explaining the intent of the slides and describing how to implement the cases, as well as how he utilized his own anecdotes and experiences. Since the implementation of the video, a few facilitators have opted for additional one-on-one instruction with one of the coauthors (e.g., by phone or on day of conference) to review the materials. Appendix D is a worksheet that is distributed at the beginning of the session to afford trainees an opportunity to reflect on a service activity in which they are engaged and that they feel is personally valuable but are unsure if it affords professional value. The facilitator can encourage participants to share what they have noted on their worksheets. Appendix E includes three case scenarios for the facilitator to introduce during PPT slides 24–26; these scenarios describe service projects implemented by trainees. These cases are analyzed during the 15-minute small-group learning segment. Each small group should optimally consist of five to seven participants. The three cases are randomly distributed amongst the groups. As an alternative, a group may choose its own real case or current project for this exercise.

In terms of evaluation, Appendix F includes questions to gauge participants' self-efficacy in transforming service work into scholarship and an assessment of how well the learning objectives have been met. Specific pre- and postworkshop questions include the following:

• Using a 5-point Likert scale (0 = *No confidence,* 4 = *Complete confidence*), indicate “How much CONFIDENCE do you have in your ability to publish your service-related work?”
• Using a 5-point Likert scale (1 = *Strongly Disagree,* 2 = *Disagree,* 3 = *Neither Agree nor Disagree,* 4 = *Agree,* 5 = *Strongly Agree*), indicate “To what extent do you agree with the following statements: (a) engaging in community-based work does not align with an academic medicine career and (b) a career in academic medicine would isolate me from my community.”
• Using a 5-point Likert scale (1 = *Strongly Disagree,* 2 = *Disagree,* 3 = *Neither Agree nor Disagree,* 4 = *Agree,* 5 = *Strongly Agree*), indicate “A career in academic medicine would (a) allow me to do work that gives me a feeling that I am serving my community and (b) allow me to engage in service work in my community of interest.”

Questions asked exclusively postworkshop included the following:

• Using a 5-point Likert scale (1 = *Strongly Disagree,* 2 = *Disagree,* 3 = *Neither Agree nor Disagree,* 4 = *Agree,* 5 = *Strongly Agree*), indicate “To what extent do you agree that the workshop learning objectives were met? (a) Define the terms *community service, community engagement,* and *service learning,* (b) describe frameworks to help achieve community service scholarship, and (c) apply steps to achieving community service scholarship: sample cases and personal stories.”

After recording their responses to the aforementioned three learning objectives, participants were then asked to answer two open-ended questions: (1) What did you like about this workshop? (2) What suggestions do you have to improve this workshop?

Reviewing the materials should take 1–2 hours, and it is highly recommended that facilitators conduct a practice session. If multiple presenters are collaborating as session leads, we also recommend a phone conversation and brief face-to-face meeting before the session begins to discuss roles/sections that each will handle. It may work best if one person serves as the main moderator to transition roles.

This workshop can be implemented among medical students and/or residents (and even junior faculty). The preferred facilitator would be a faculty member with an MD or DO degree and experience in service-based work and/or scholarship. One or two facilitators can implement this workshop. If two cofacilitators do, an effort should be made for them to meet and divide the different sections of the presentation equally in an integrative fashion. The optimal timing for the workshop is 75 minutes. This can be shortened by having students complete the preworkshop questions and worksheet prior to the workshop and/or by replacing the small-group discussion of cases with a large-group discussion.

## Results

At five regional conferences between June 2016 and December 2016, the workshop was facilitated by a total of six presenters (four single presenters and one pair of cofacilitators) at various levels in their careers: assistant professor (three), associate professor (one), and full professor (two). All facilitators were faculty and/or had publications related to service work or community-engaged participatory research.

One hundred thirty-nine trainees participated in the workshop. The 139 respondents were a diverse sample—72 (51.8%) identified as women; 60 (43.2%) as men; 30 (21.6%) as lesbian, gay, bisexual, or queer; 38 (27.3%) as Hispanic/Latino; 36 (25.9%) as white; 40 (28.8%) as African-American/black; 28 (20.1%) as Asian; and two (1.4%) as American Indian. There were 124 medical student respondents and 15 resident respondents who were training in Washington, DC, and 13 different states.

One hundred eleven (79.9%) learners responded to the pre- and postworkshop questions. In comparing responses pre- and postworkshop using paired-samples *t* tests, there was a statistically significant increase in participants' confidence to publish service-related work (preworkshop *M* = 1.98 vs. postworkshop *M* = 2.87, *p* < .001), and their belief that a career in academic medicine would both allow them to do work that gives them a feeling they are serving their community (3.88 vs. 4.25, *p* < .001) and to engage in service work in their community of interest (3.82 vs. 4.26, *p* < .001). Participants were statistically less likely to agree that engaging in community-based work did not align with an academic medicine career (2.04 vs. 1.75, *p* < .01) and that a career in academic medicine would isolate them from their community (2.24 vs. 1.88, *p* < .001).

Seventy-four (53.2%) learners responded to the question, “To what extent do you agree that the workshop learning objectives were met?” More than 97% agreed or strongly agreed that the learning objectives were met. Their responses are summarized in the Table.

**Table.** Learner Responses to the Question, “To What Extent Do You Agree That the Workshop Learning Objectives Were Met?” (*N* = 74)

Objective	No. (%)				
	Strongly Agree	Agree	Neither Agree nor Disagree	Disagree	Strongly Disagree
Define the terms *community service, community engagement*, and *service learning.*	49 (66.2)	25 (33.8)	0 (0)	0 (0)	0 (0)
Describe frameworks to help achieve community service scholarship.	46 (62.2)	27 (36.5)	1 (1.3)	0 (0)	0 (0)
Apply steps to achieving service scholarship: mock cases and personal stories.	47 (63.6)	26 (35.1)	1 (1.3)	0 (0)	0 (0)

We organized participants' comments by learning objectives for the questions, “What did you like about this workshop?” and “What suggestions do you have to improve this workshop?” Comments for the workshop were overall positive, with a few suggestions for improvement. Specific comments, as related to each of the learning objectives, are provided below:

What did you like about this workshop?

• Objective 1: Define the terms *community service, community engagement,* and *service learning.*
  ○ “I discovered that it is possible to transform service work into published work. This workshop provided very useful information about framing service learning.”
  ○ “Describes ways to transform community services into academic-related activities, which eventually leads to a career in academic medicine.”
• Objective 2: Describe frameworks to help achieve community service scholarship.
  ○ “I really appreciated this workshop. Service is a huge priority of mine, so I was very happy to learn about how to integrate this into building an academic career.”
  ○ “Great talk on community service. Now I feel like I have a better understanding on how to make my interests work for me. I want to keep doing my community and global work and want them to be considered as credible accomplishments. This session helped me understand that this is possible.”
• Objective 3: Apply steps to achieving service scholarship: sample cases and personal stories.
  ○ “Great workshop! Enjoyed getting hands on cases to think about subject. Facts and figures given were truly appreciated. Now I know to turn my work into actual scholarship and publication AND how this type of work could impact my career development/progression.”
  ○ “There are many service opportunities at my institution that don't incorporate scholarship, and it was great to get concrete examples of how to do that.”
  ○ “I appreciated the case examples + how those became publications. It solidified how I may achieve service scholarship.”

What suggestions do you have to improve this workshop?

• Objective 1: Define the terms *community s* *ervice, community engagement,* and *service learning.*
  ○ There were no responses relevant to objective 1 to improve this workshop.
• Objective 2: Describe frameworks to help achieve community service scholarship.
  ○ “Practical application exercises allow for active learning. A suggestion would be to explain certain models presented. As a medical student, most are not well acquainted with the Kern model. Maybe an explanation of the model and how to use it would be very helpful. The workshops clarified different types of scholarship, which often is unknown to medical students.”
  ○ “Would love more workshop and specific listing of modes of translating service projects to publication. More ‘How?’”
• Objective 3: Apply steps to achieving service scholarship: sample cases and personal stories.
  ○ “It was nice to hear about how service learning can be scholarship, but I wanted to hear some specific examples of how to do it.”
  ○ “I would have liked more examples/explanations on how to transform student ideas/projects into scholarship/publishable units.”

Overall, participants reported that the workshop heightened their awareness of the relevance of service work and scholarship to their career trajectory and of how to transform service work into academic-related scholarship. Over 95% of participants strongly agreed or agreed that the learning objectives were met. Through pre- and postworkshop evaluation questions, learners expressed an increased confidence in the ability to publish service-related work, were less likely to feel that service work did not align with an academic medicine career, and were more likely to feel that a career in academic medicine would allow them to engage in service work in their community of interest. The responses to the open-ended questions showed that for our participants, service work was part of their personal and professional identity and that they greatly appreciated increased awareness of how to align this passion with an academic medicine career. Moreover, they desired a deeper appreciation of frameworks to help transform their service work into scholarship.

## Discussion

The majority of participants reported that they gained an appreciation of the importance of transforming service into service scholarship, as well as of how to apply frameworks to accomplish this. Additionally, the case scenarios and subsequent examples of related publications gave students a practical example of achieving service scholarship.

The majority of participants felt the workshop gave them an outline of how to achieve service scholarship, but a few participants desired a deeper appreciation of the frameworks (i.e., the Kern and Cené models) highlighted in the workshop and examples of additional frameworks. To help facilitate a deeper appreciation of the Kern and Cené models, we modified the discussion guide to provide specific examples of how either model can be used for the three case scenarios. Additionally, we have noted that the facilitator should emphasize that the workshop aims to provide a heightened awareness of using a model to achieve service scholarship, as well as highlighting resources that promote a more complete understanding of each model's application. We also added additional PPT slides to stress that there are numerous models that can be considered, and we highlighted the SMART model and collaborative community health research, the latter of which has been featured in prior *MedEdPORTAL* publications.^19,28^

Most importantly, the facilitator should accentuate the importance of selecting an appropriate framework for a project. Facilitators should acknowledge that this ought to be done right from the beginning of conceptualizing a project. In discussing their own service work, facilitators should state whether they have utilized one of the aforementioned frameworks (e.g., the Kern model, the SMART model etc.) or a different one. An alternate approach is to have participants describe their service work and any frameworks they have considered. Participants can be encouraged to provide a description of their service project to the facilitator prior to the workshop or during the Q&A session, thus affording personalized guidance. However, this approach would lengthen the time allotment of the workshop.

It is important to note the limitations of our assessment. Our workshop is a brief, onetime intervention, and a sustained awareness of how to complete community service scholarship or how community service scholarship facilitates an academic career requires reinforcement, such as additional teaching, role modeling, or mentoring. Moreover, our assessment was self-reported and conducted right before and right after the workshop. A sustained change in self-efficacy would require reassessment at later time points (e.g., 6 months, 12 months, etc.). A followup assessment could also inquire about use and choice of frameworks to facilitate scholarship and quantify a change in scholarly productivity (e.g., number of presentations or publications).

In implementing this workshop, consideration should be given to identifying a facilitator who has experience in conducting service work and service scholarship. As described in the presentation, service experience can entail work performed on the local, regional, international, or institutional level. The facilitator should have experience in working with medical students or residents or with the target workshop audience. Additionally, if the intent of the workshop is to target certain groups such as those underrepresented or invisible in academia (e.g., underrepresented minorities), consideration should be given to identifying a facilitator of the same identity or with practical experience in serving the same community. The literature indicates that these groups lack and desire concordant role models and mentors, especially when considering academic careers.^3,5,8^ Although such attributes are not necessary for a facilitator, they do further substantiate the facilitator's identity as a role model and potential advisor or mentor with practical experience in guiding trainees in completing scholarship.

Dental faculty members have collaborated with us to modify the workshop to enhance dental students' and dental hygienist students' self-efficacy in transforming service work into scholarship. The modifications have consisted of including data and statistics on dental trainees' interest and level of service work along their educational journey and altering the cases to be more relatable to dental trainees. In a similar fashion, the workshop can be modified for an audience seeking other health professional degrees, such as physician assistant, nursing, or pharmacy.

## Appendices

A. PowerPoint Presentation.pptx
B. Slide Instructions.docx
C. Train the Trainer Workshop Video.mp4
D. Worksheet.doc
E. Case Scenarios.doc
F. Evaluation Form.doc All appendices are peer reviewed as integral parts of the Original Publication.
