# Supplementary material for: Helping Trainees Develop Scholarship in Academic Medicine From Community Service
Source: MedEdPORTAL. 2017 Dec 14;13:10659. doi: 10.15766/mep_2374-8265.10659 (PMC6338162; doi:10.15766/mep_2374-8265.10659)
Supplement: Supplementary file 1 — A. PowerPoint Presentation.pptx B. Slide Instructions.docx C. Train the Trainer Workshop Video.mp4 D. Worksheet.doc E. Case Scenarios.doc F. Evaluation Form.doc [file mep-13-10659-s001.zip › A. PowerPoint Presentation.pptx]

## Slide 1
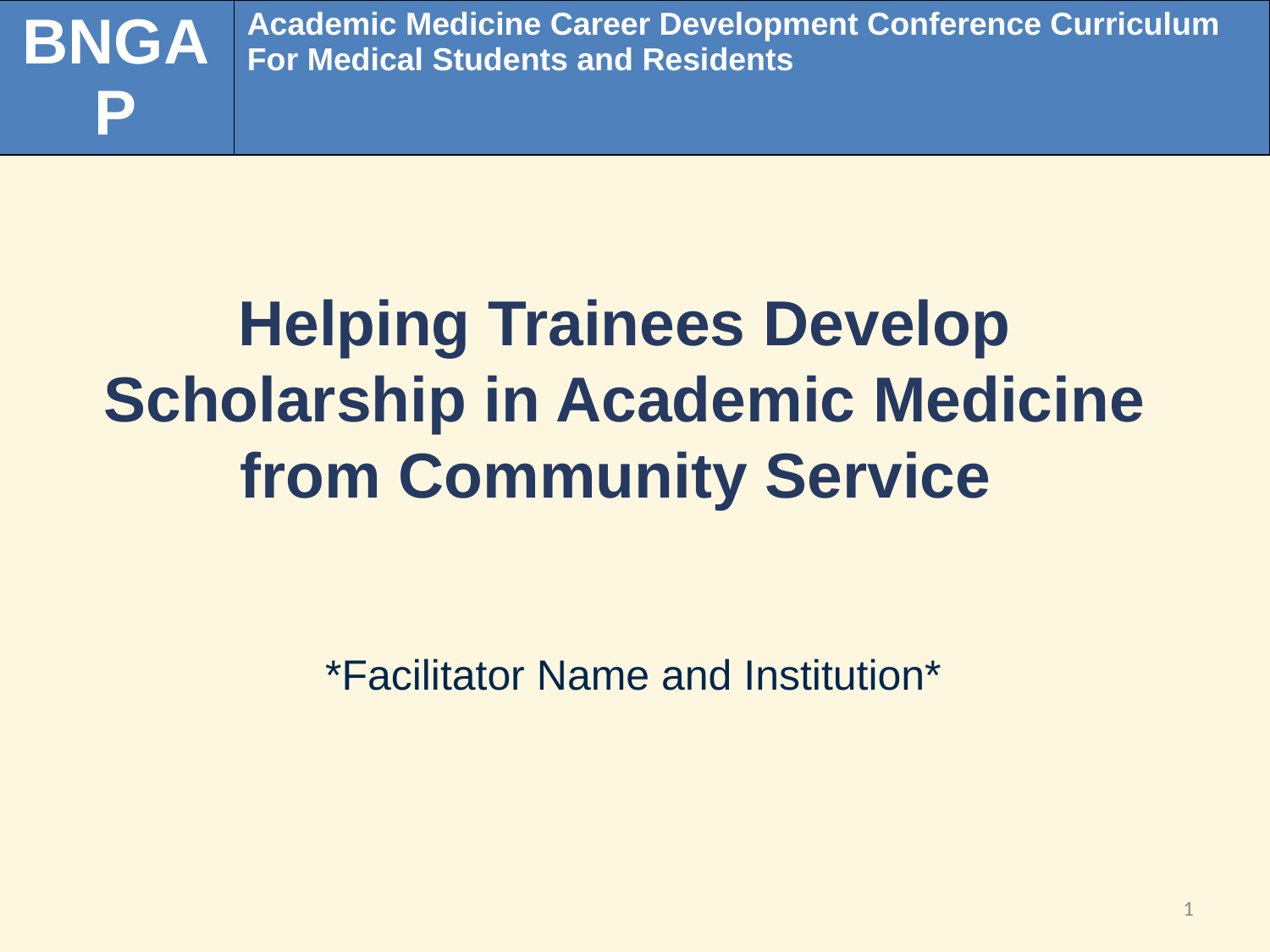

Helping Trainees Develop Scholarship in Academic Medicine from Community Service
*Facilitator Name and Institution*
1

## Slide 2
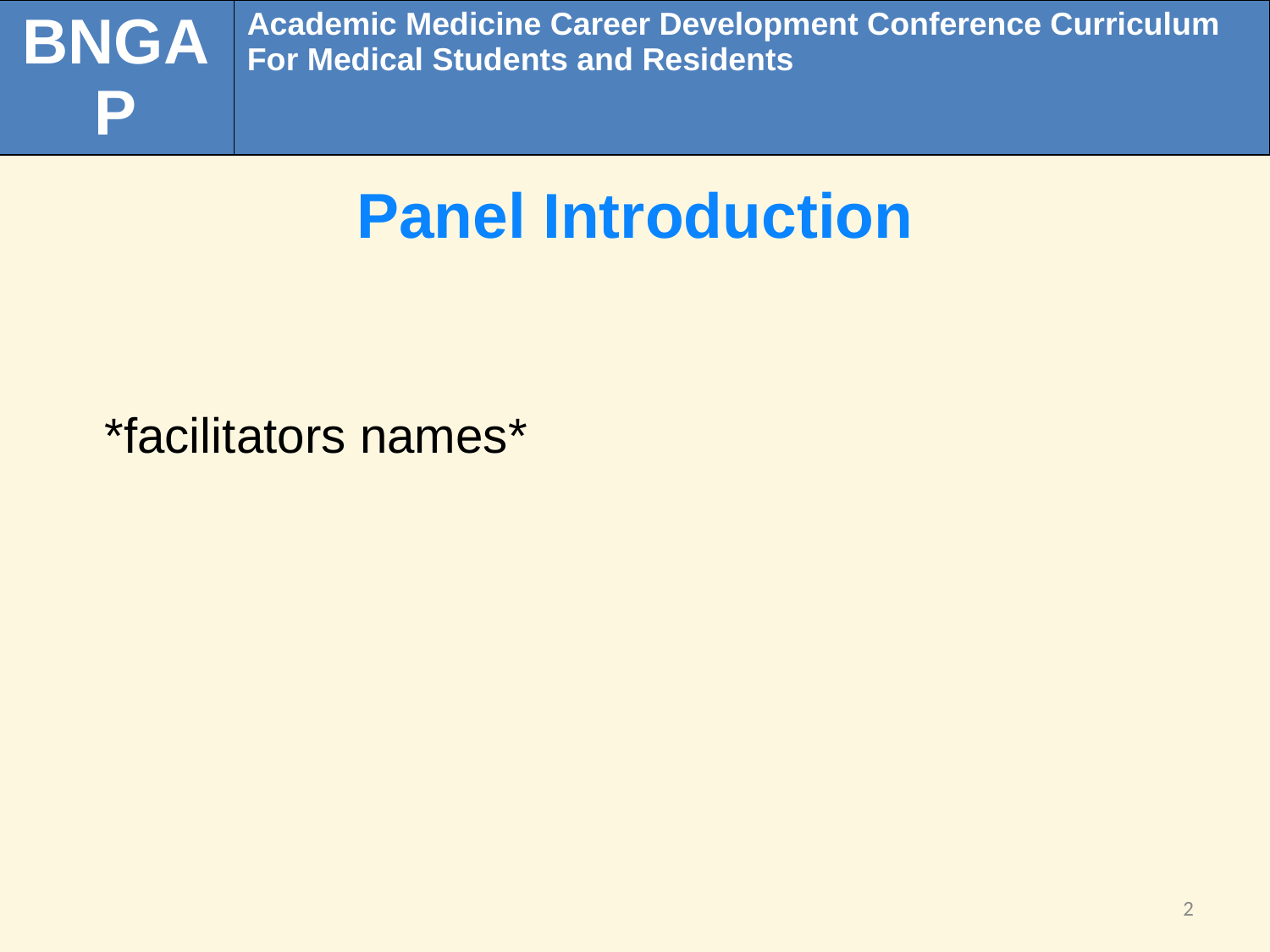

# Panel Introduction
*facilitators names*
2

## Slide 3
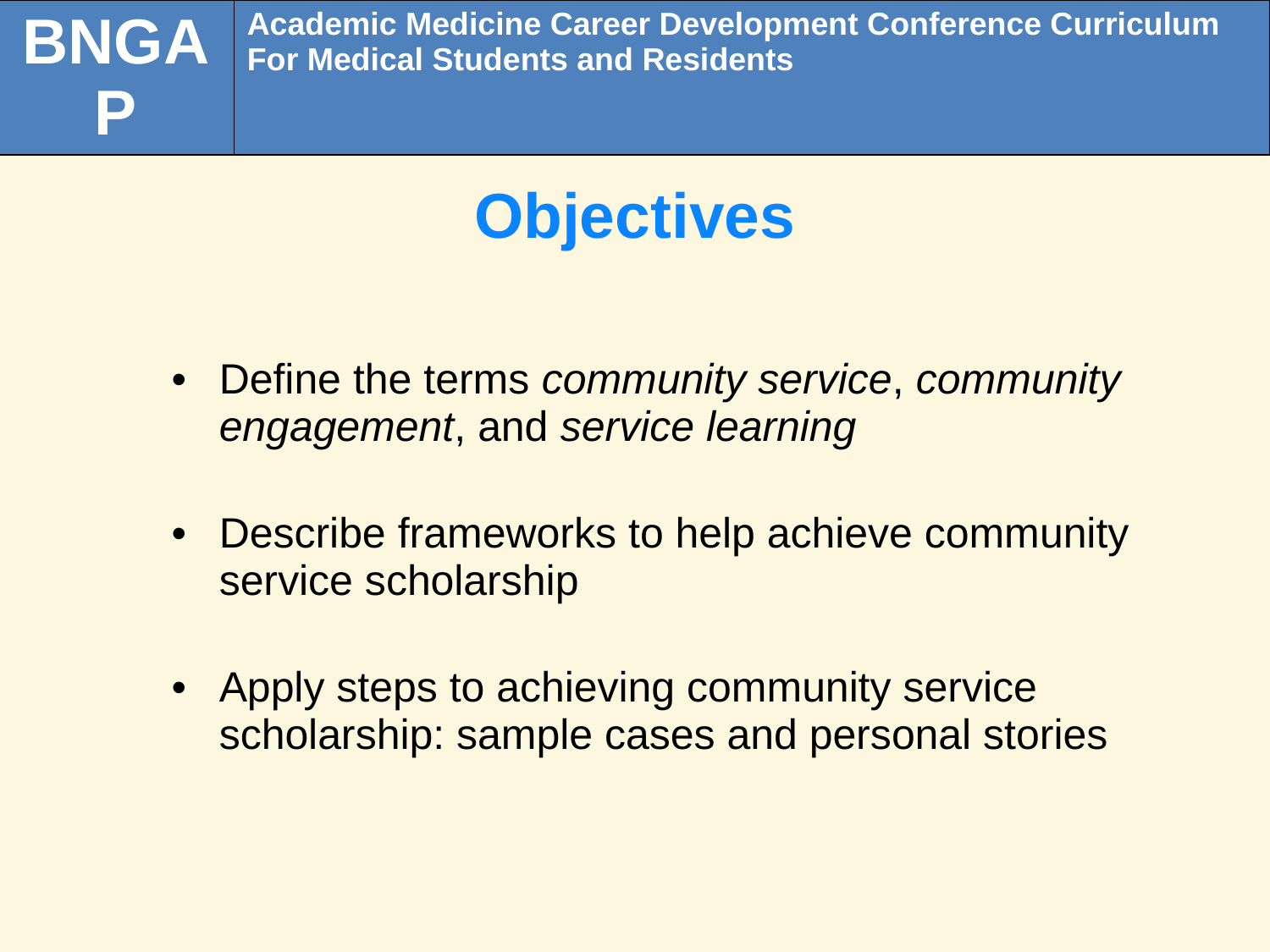

# Objectives
| | Define the terms community service, community engagement, and service learning |
| --- | --- |
| | Describe frameworks to help achieve community service scholarship |
| | Apply steps to achieving community service scholarship: sample cases and personal stories |

## Slide 4
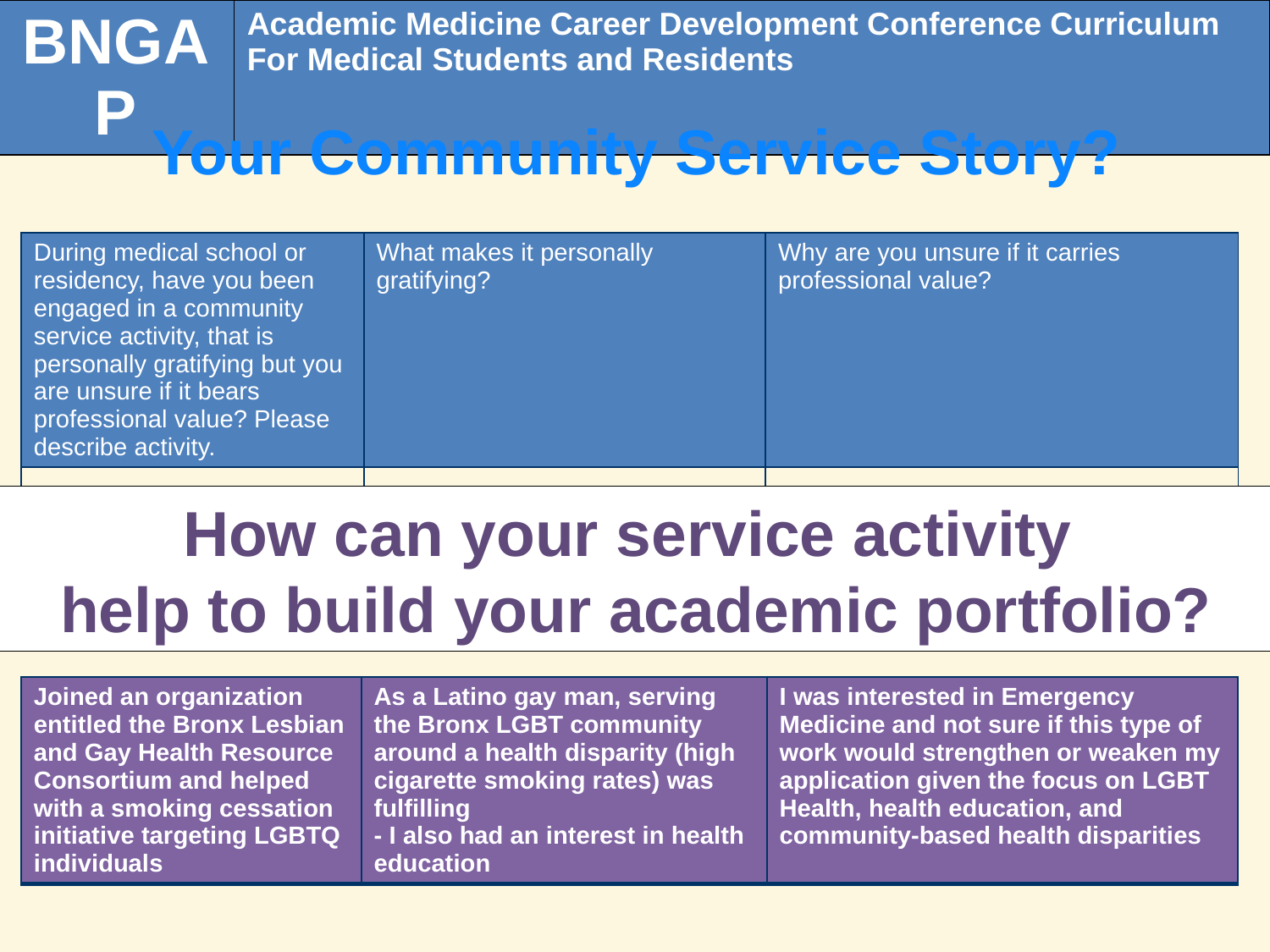

# Your Community Service Story?
| During medical school or residency, have you been engaged in a community service activity, that is personally gratifying but you are unsure if it bears professional value? Please describe activity. | What makes it personally gratifying? | Why are you unsure if it carries professional value? |
| --- | --- | --- |
| | | |
How can your service activity
help to build your academic portfolio?
| Joined an organization entitled the Bronx Lesbian and Gay Health Resource Consortium and helped with a smoking cessation initiative targeting LGBTQ individuals | As a Latino gay man, serving the Bronx LGBT community around a health disparity (high cigarette smoking rates) was fulfilling - I also had an interest in health education | I was interested in Emergency Medicine and not sure if this type of work would strengthen or weaken my application given the focus on LGBT Health, health education, and community-based health disparities |
| --- | --- | --- |

## Slide 5
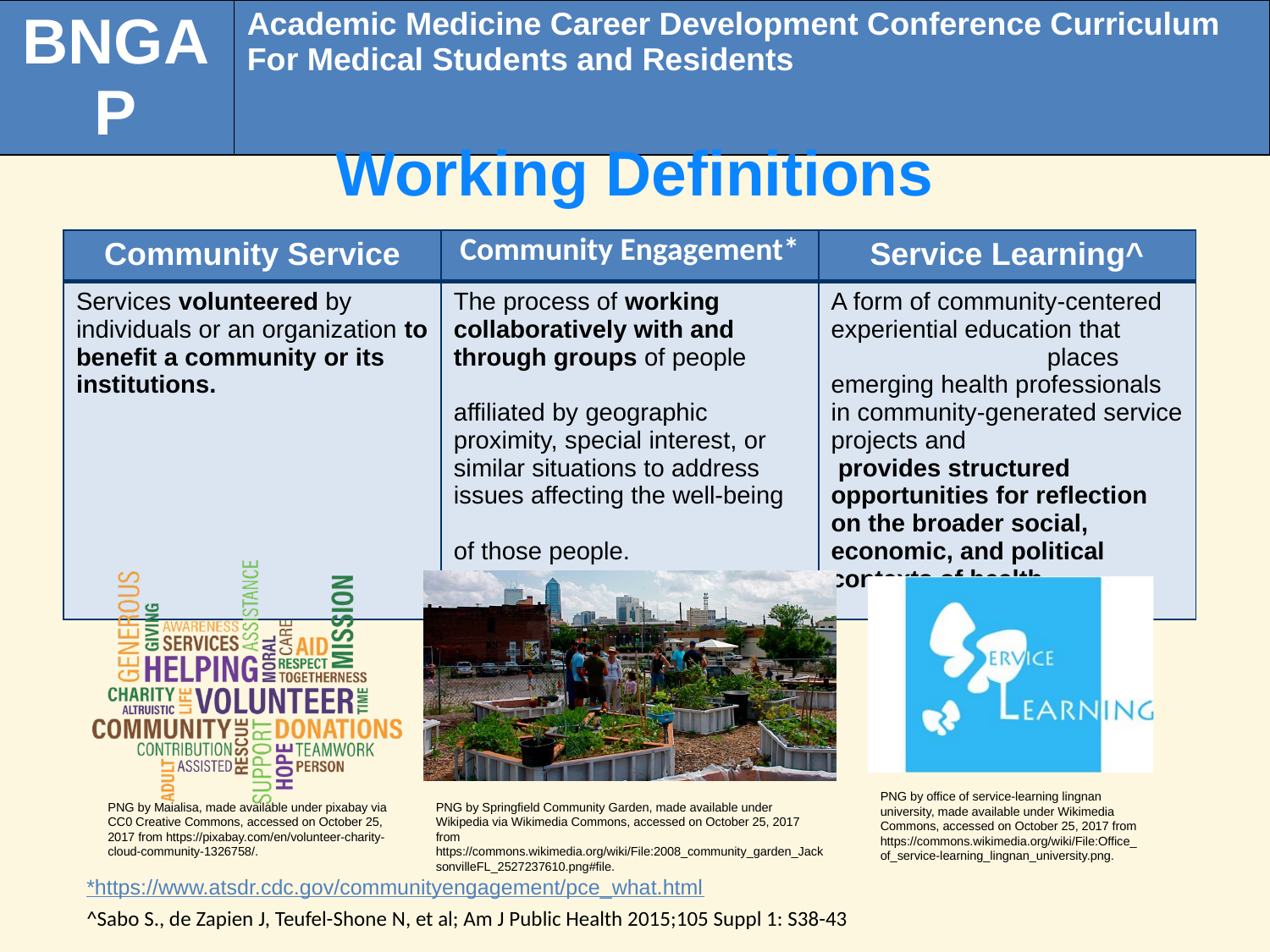

# Working Definitions
| Community Service | Community Engagement\* | Service Learning^ |
| --- | --- | --- |
| Services volunteered by individuals or an organization to benefit a community or its institutions. | The process of working collaboratively with and through groups of people affiliated by geographic proximity, special interest, or similar situations to address issues affecting the well-being of those people. | A form of community-centered experiential education that places emerging health professionals in community-generated service projects and provides structured opportunities for reflection on the broader social, economic, and political contexts of health. |
PNG by office of service-learning lingnan university, made available under Wikimedia Commons, accessed on October 25, 2017 from https://commons.wikimedia.org/wiki/File:Office_of_service-learning_lingnan_university.png.
PNG by Maialisa, made available under pixabay via CC0 Creative Commons, accessed on October 25, 2017 from https://pixabay.com/en/volunteer-charity-cloud-community-1326758/.
PNG by Springfield Community Garden, made available under Wikipedia via Wikimedia Commons, accessed on October 25, 2017 from https://commons.wikimedia.org/wiki/File:2008_community_garden_JacksonvilleFL_2527237610.png#file.
*https://www.atsdr.cdc.gov/communityengagement/pce_what.html
^Sabo S., de Zapien J, Teufel-Shone N, et al; Am J Public Health 2015;105 Suppl 1: S38-43

## Slide 6
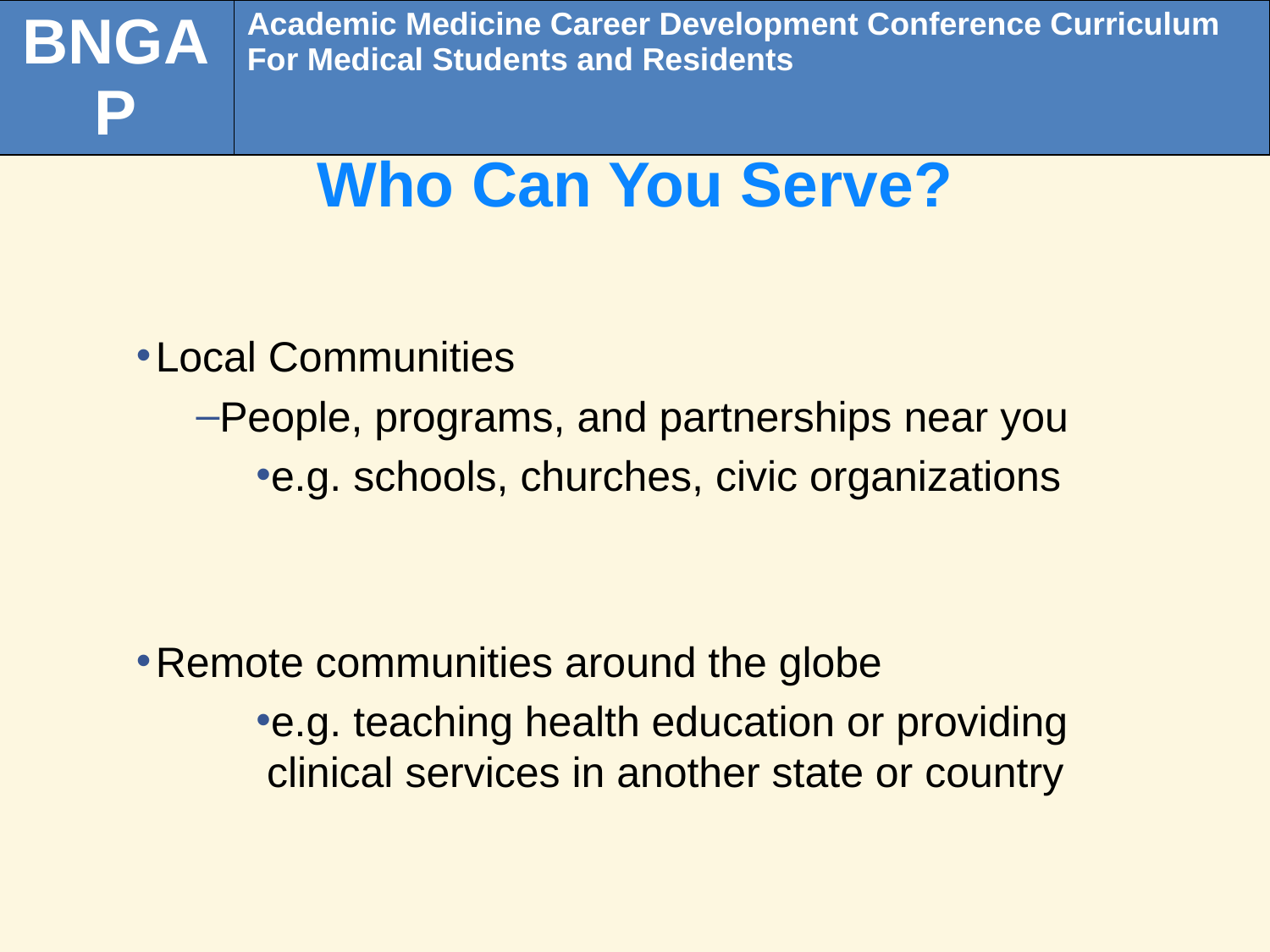

# Who Can You Serve?
Local Communities
People, programs, and partnerships near you
e.g. schools, churches, civic organizations
Remote communities around the globe
e.g. teaching health education or providing clinical services in another state or country

## Slide 7
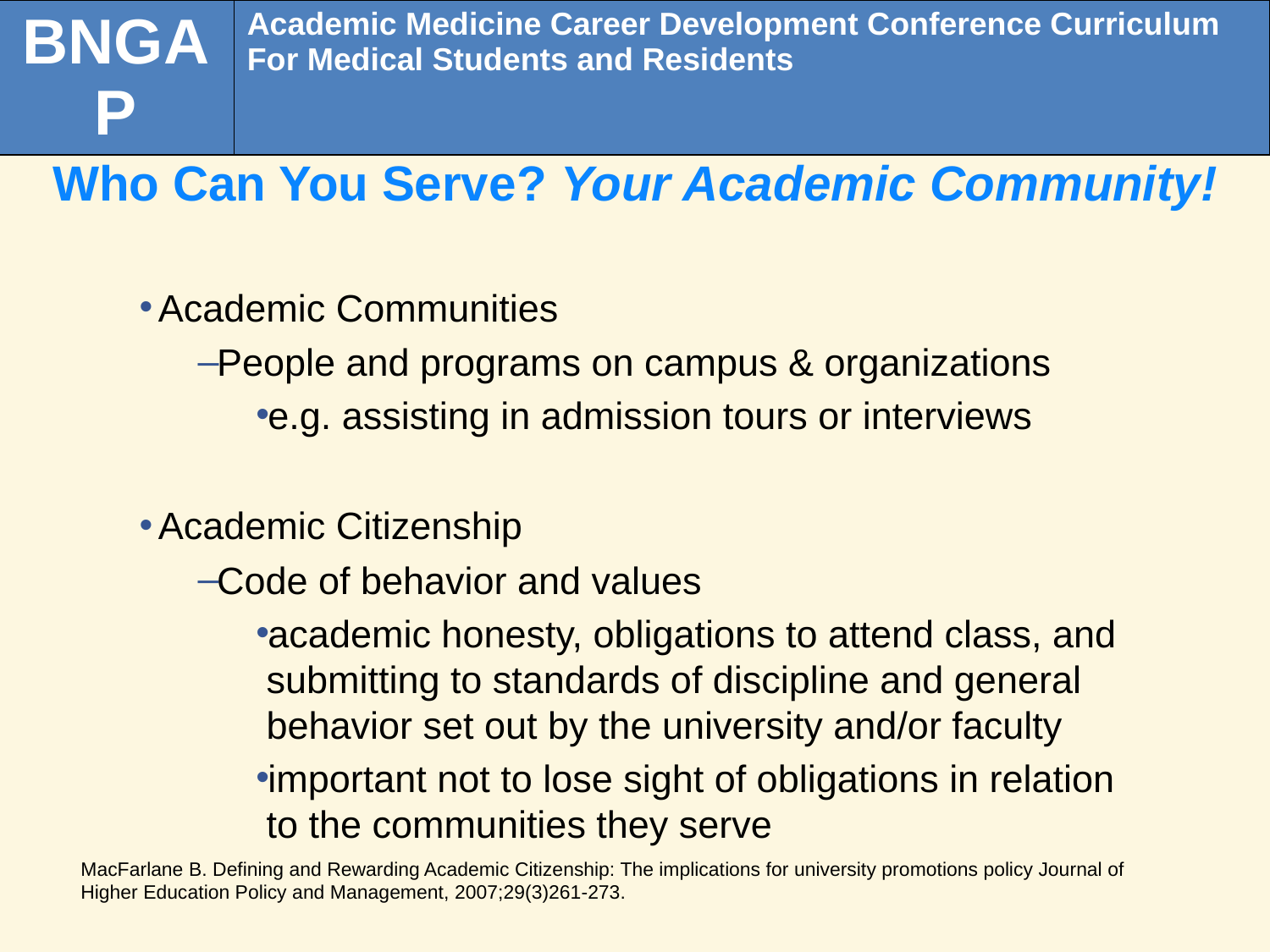

# Who Can You Serve? Your Academic Community!
Academic Communities
People and programs on campus & organizations
e.g. assisting in admission tours or interviews
Academic Citizenship
Code of behavior and values
academic honesty, obligations to attend class, and submitting to standards of discipline and general behavior set out by the university and/or faculty
important not to lose sight of obligations in relation to the communities they serve
MacFarlane B. Defining and Rewarding Academic Citizenship: The implications for university promotions policy Journal of Higher Education Policy and Management, 2007;29(3)261-273.

## Slide 8
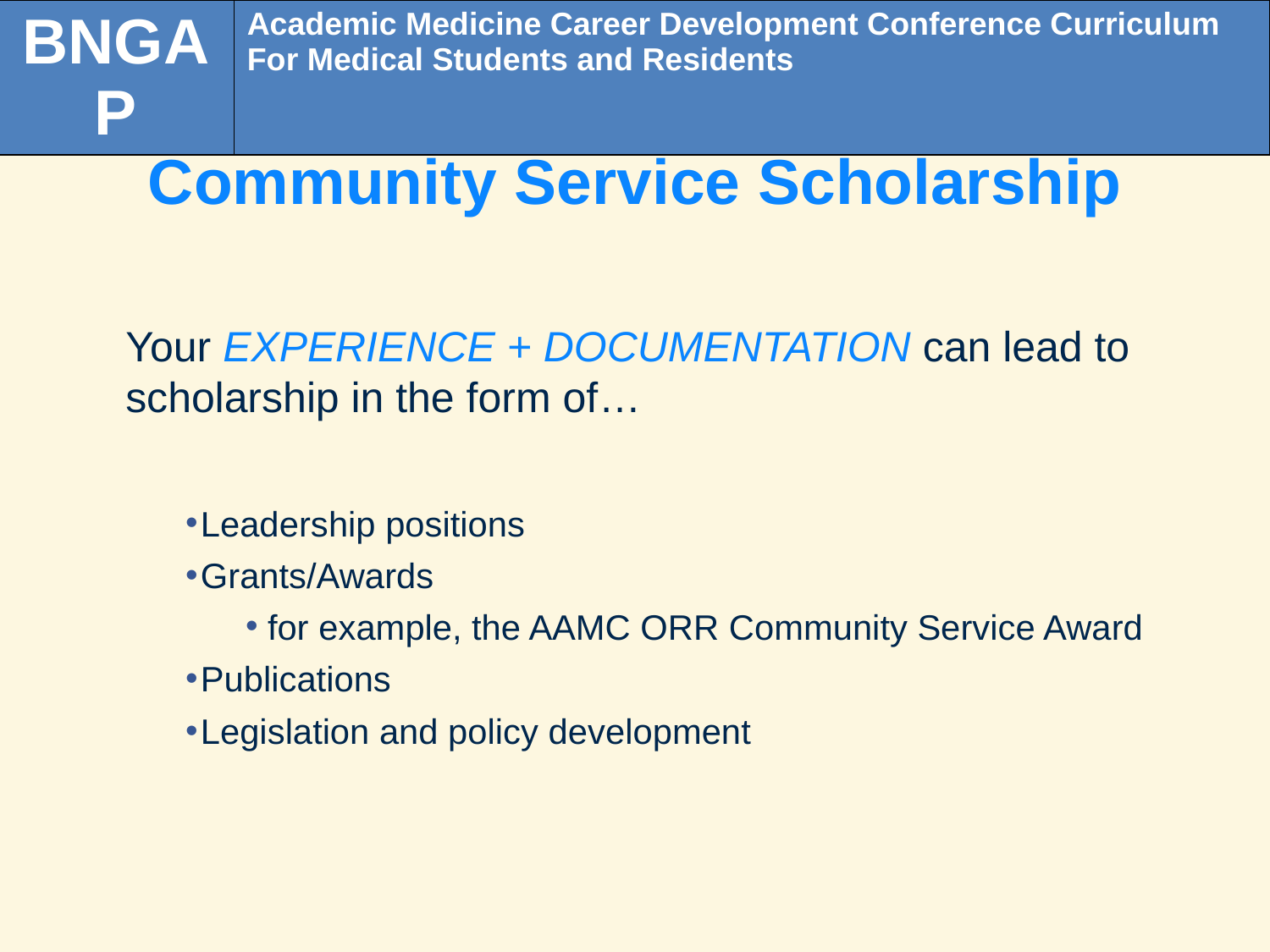

# Community Service Scholarship
Your EXPERIENCE + DOCUMENTATION can lead to scholarship in the form of…
Leadership positions
Grants/Awards
 for example, the AAMC ORR Community Service Award
Publications
Legislation and policy development

## Slide 9
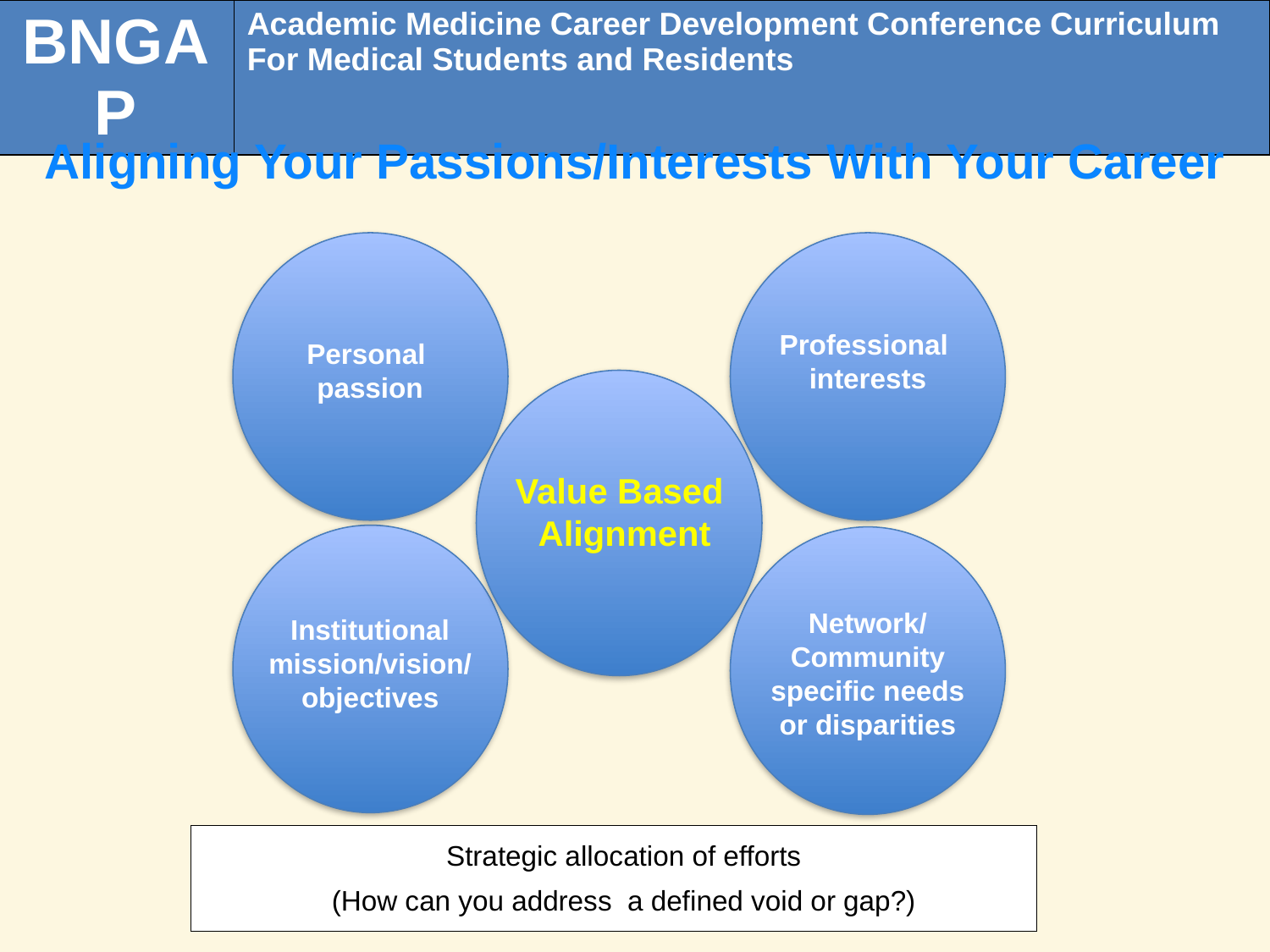

Aligning Your Passions/Interests With Your Career
Professional
interests
Personal
passion
Value Based
Alignment
Network/
Community specific needs or disparities
Institutional mission/vision/ objectives
Strategic allocation of efforts
(How can you address a defined void or gap?)

## Slide 10
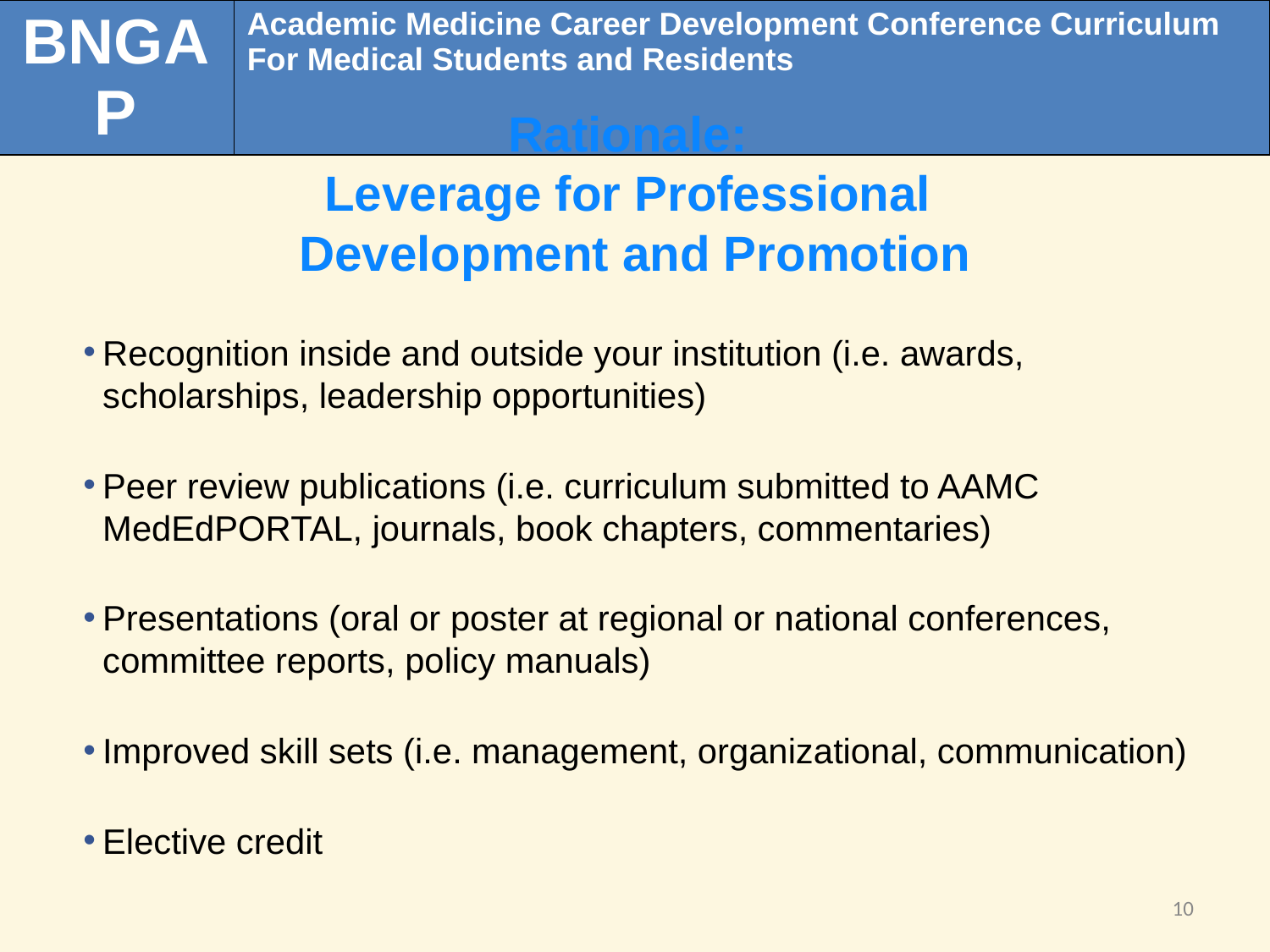

# Rationale: Leverage for Professional Development and Promotion
Recognition inside and outside your institution (i.e. awards, scholarships, leadership opportunities)
Peer review publications (i.e. curriculum submitted to AAMC MedEdPORTAL, journals, book chapters, commentaries)
Presentations (oral or poster at regional or national conferences, committee reports, policy manuals)
Improved skill sets (i.e. management, organizational, communication)
Elective credit
10

## Slide 11
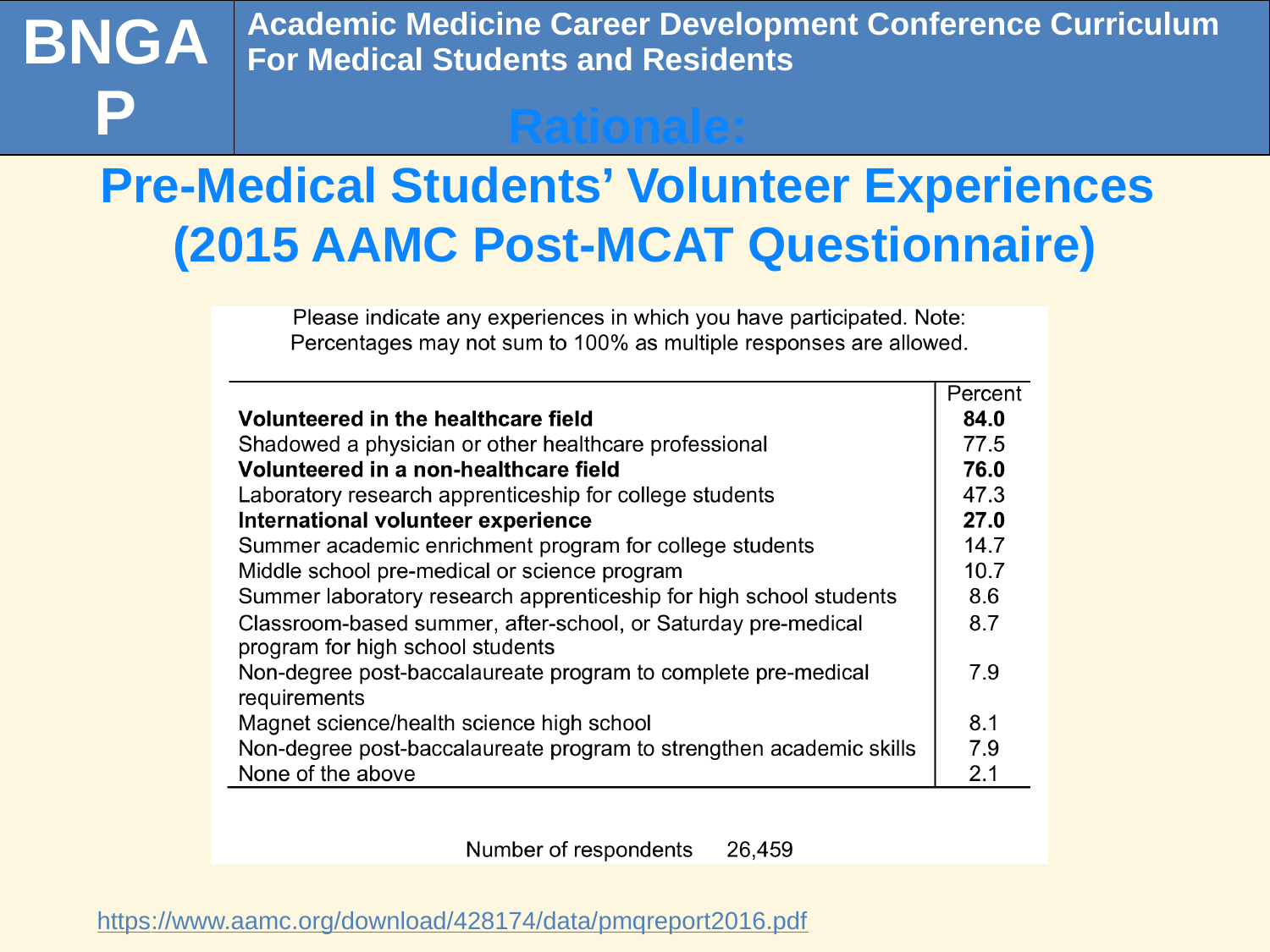

# Rationale: Pre-Medical Students’ Volunteer Experiences (2015 AAMC Post-MCAT Questionnaire)
https://www.aamc.org/download/428174/data/pmqreport2016.pdf

## Slide 12
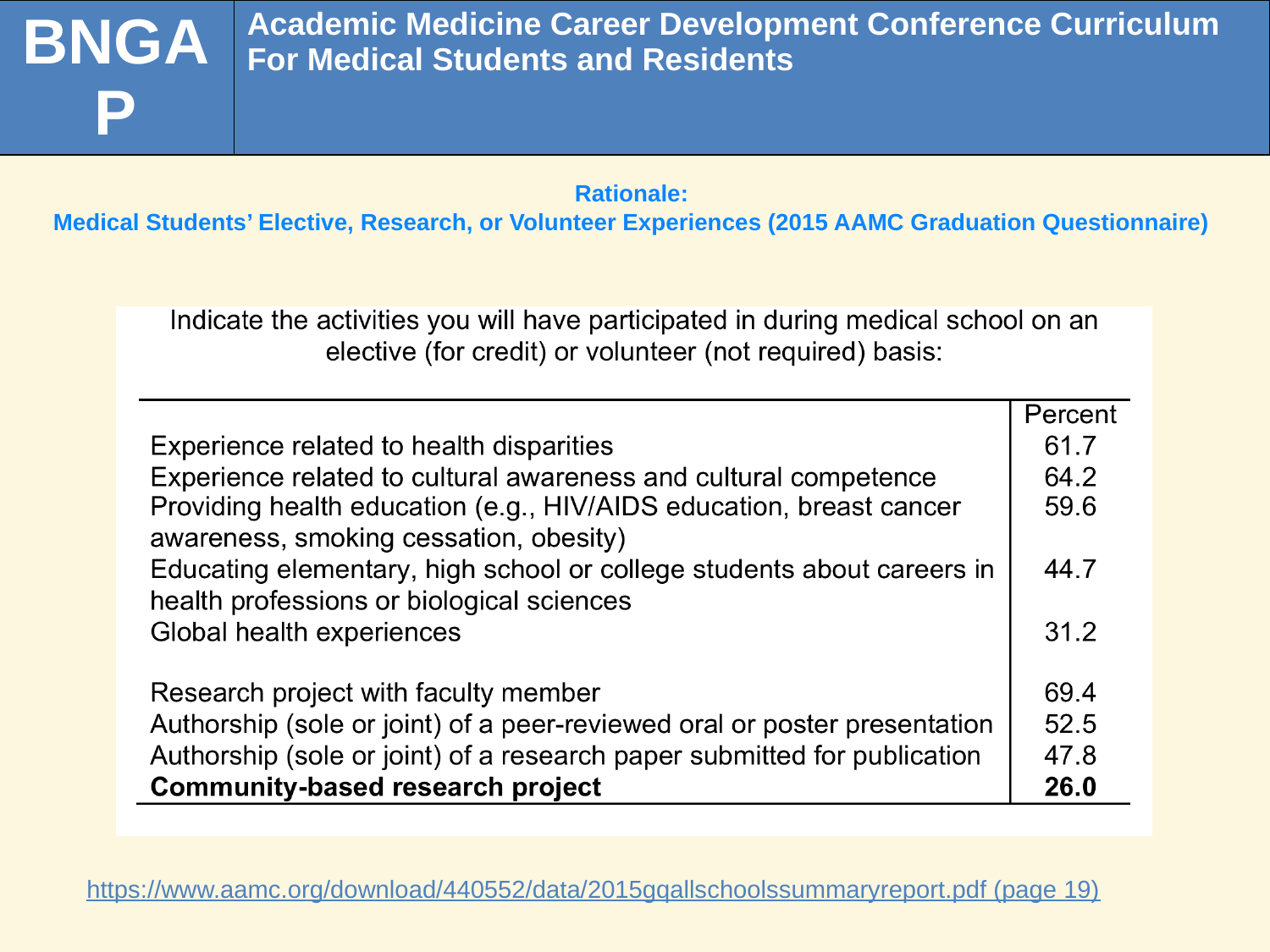

# Rationale: Medical Students’ Elective, Research, or Volunteer Experiences (2015 AAMC Graduation Questionnaire)
https://www.aamc.org/download/440552/data/2015gqallschoolssummaryreport.pdf (page 19)

## Slide 13
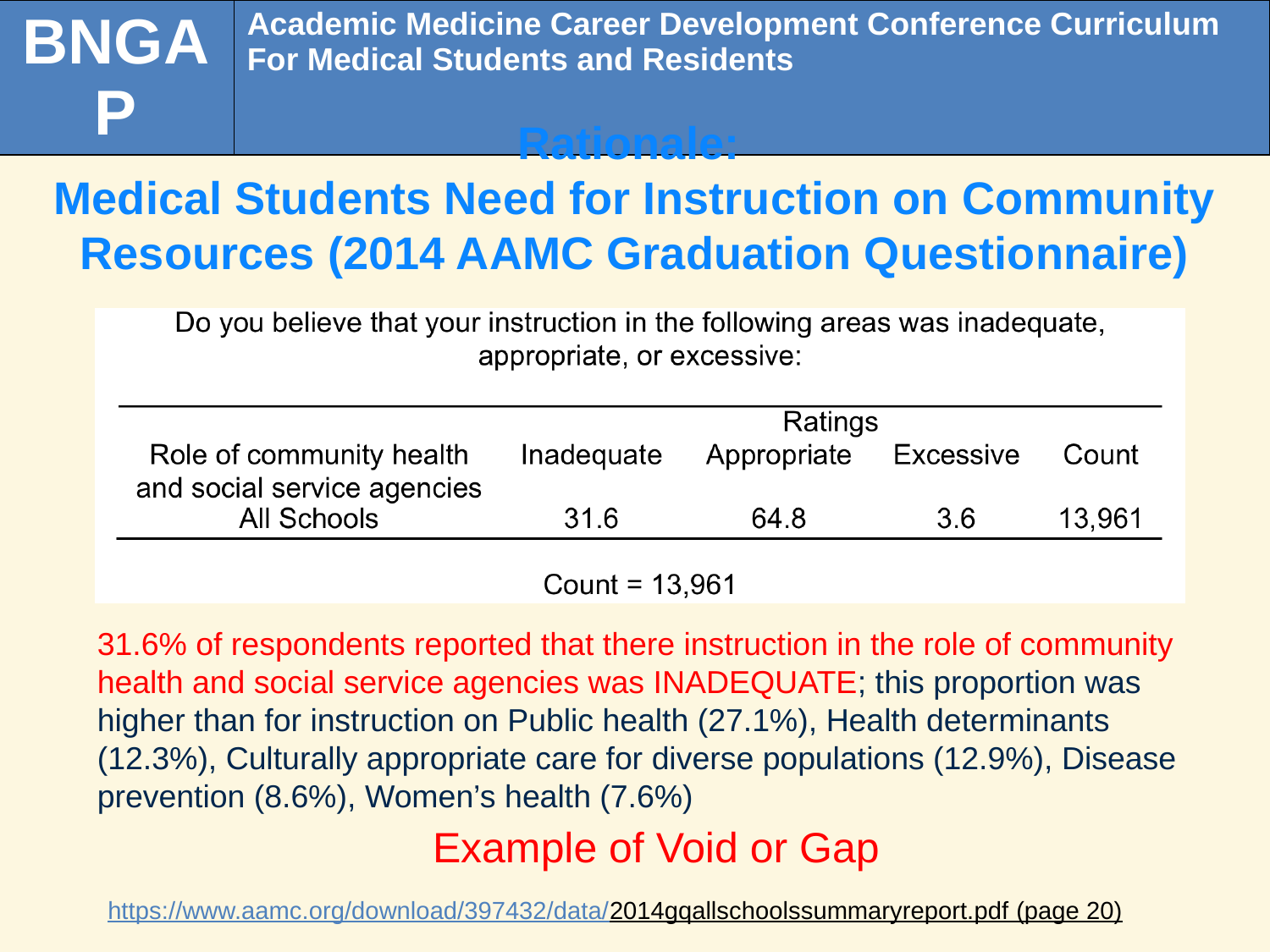

# Rationale: Medical Students Need for Instruction on Community Resources (2014 AAMC Graduation Questionnaire)
31.6% of respondents reported that there instruction in the role of community health and social service agencies was INADEQUATE; this proportion was higher than for instruction on Public health (27.1%), Health determinants (12.3%), Culturally appropriate care for diverse populations (12.9%), Disease prevention (8.6%), Women’s health (7.6%)
Example of Void or Gap
https://www.aamc.org/download/397432/data/2014gqallschoolssummaryreport.pdf (page 20)

## Slide 14
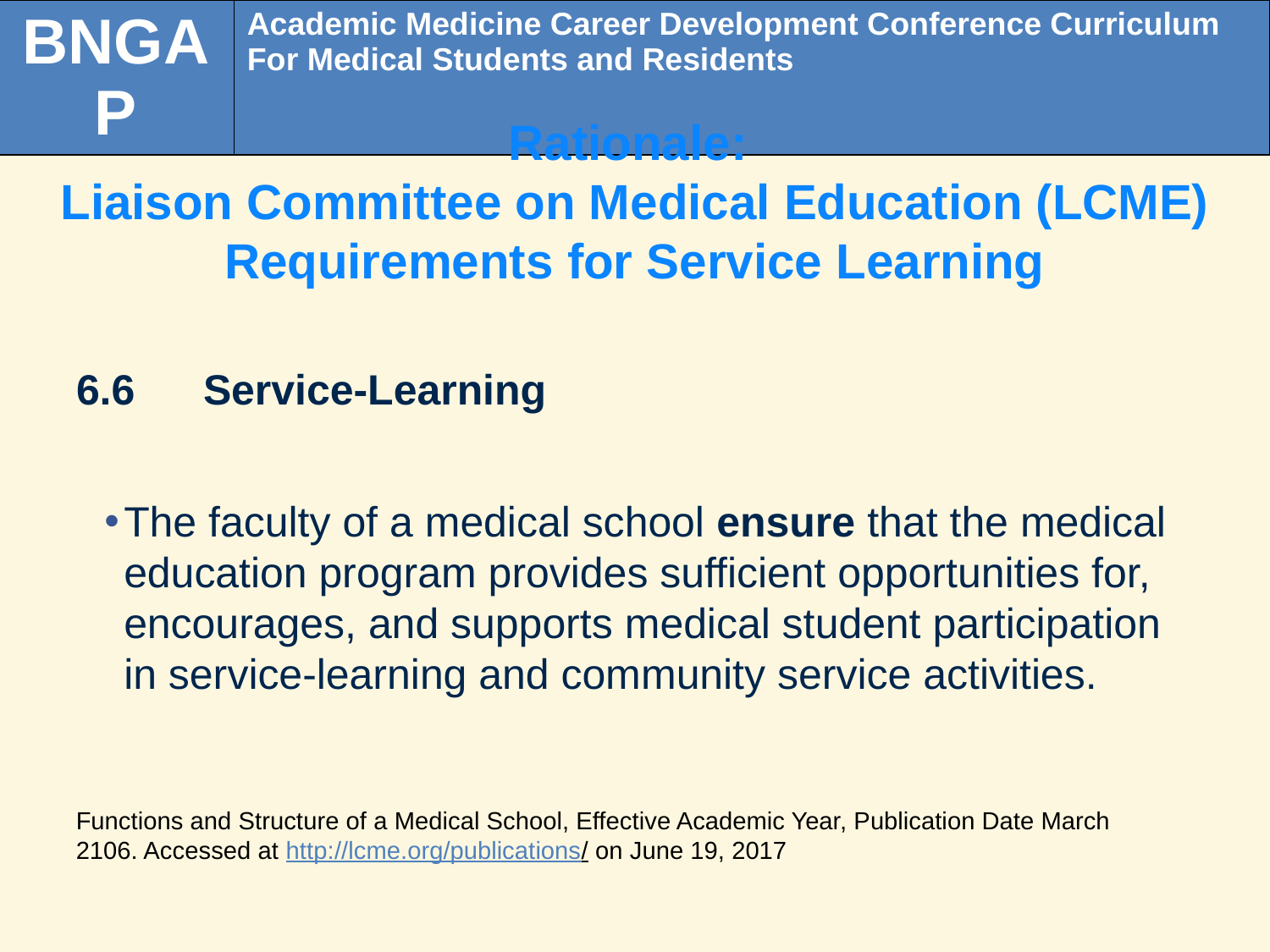

# Rationale: Liaison Committee on Medical Education (LCME) Requirements for Service Learning
6.6 	Service-Learning
The faculty of a medical school ensure that the medical education program provides sufficient opportunities for, encourages, and supports medical student participation in service-learning and community service activities.
Functions and Structure of a Medical School, Effective Academic Year, Publication Date March 2106. Accessed at http://lcme.org/publications/ on June 19, 2017

## Slide 15
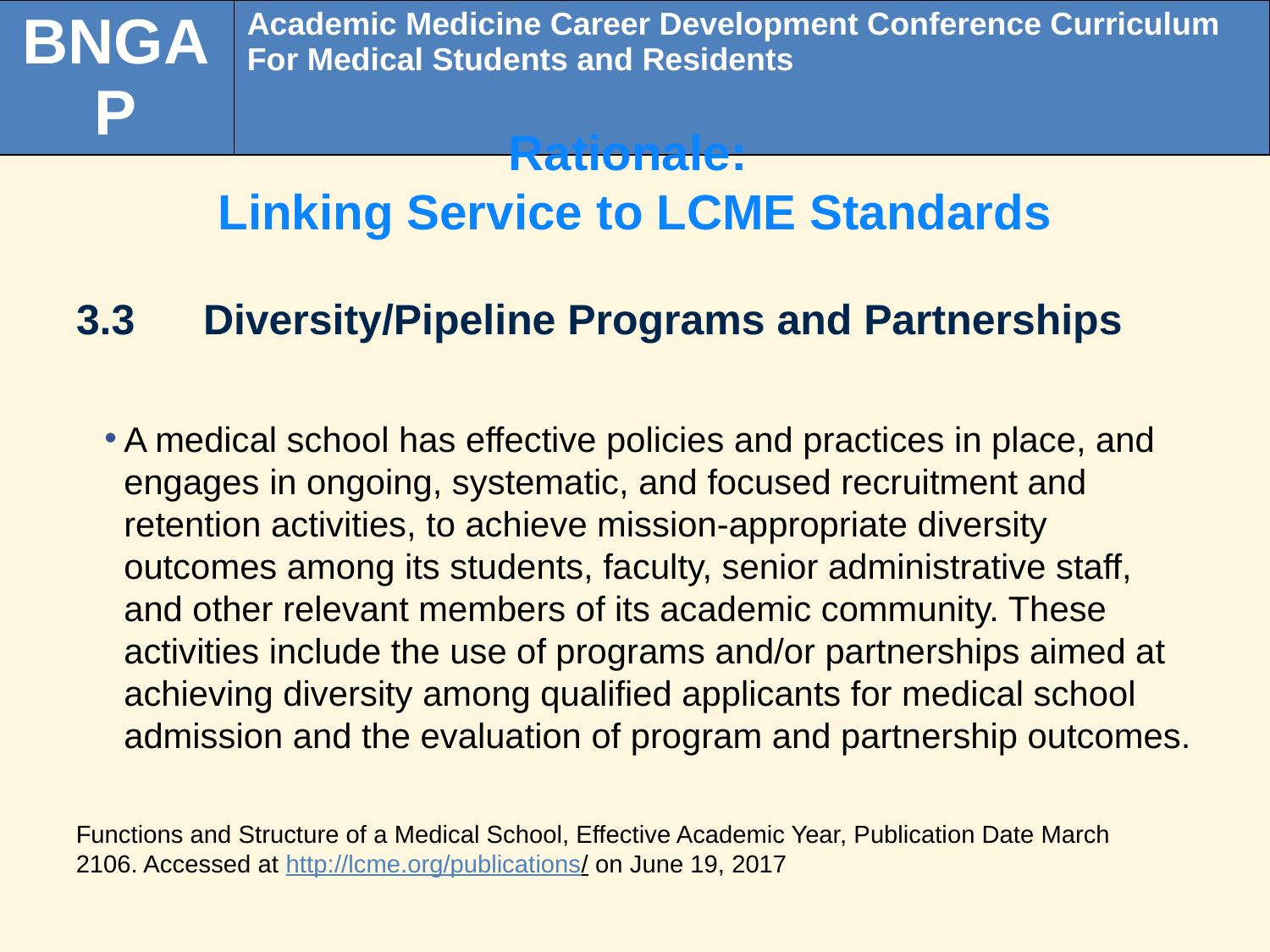

Rationale:
Linking Service to LCME Standards
3.3 	Diversity/Pipeline Programs and Partnerships
A medical school has effective policies and practices in place, and engages in ongoing, systematic, and focused recruitment and retention activities, to achieve mission-appropriate diversity outcomes among its students, faculty, senior administrative staff, and other relevant members of its academic community. These activities include the use of programs and/or partnerships aimed at achieving diversity among qualified applicants for medical school admission and the evaluation of program and partnership outcomes.
Functions and Structure of a Medical School, Effective Academic Year, Publication Date March 2106. Accessed at http://lcme.org/publications/ on June 19, 2017

## Slide 16
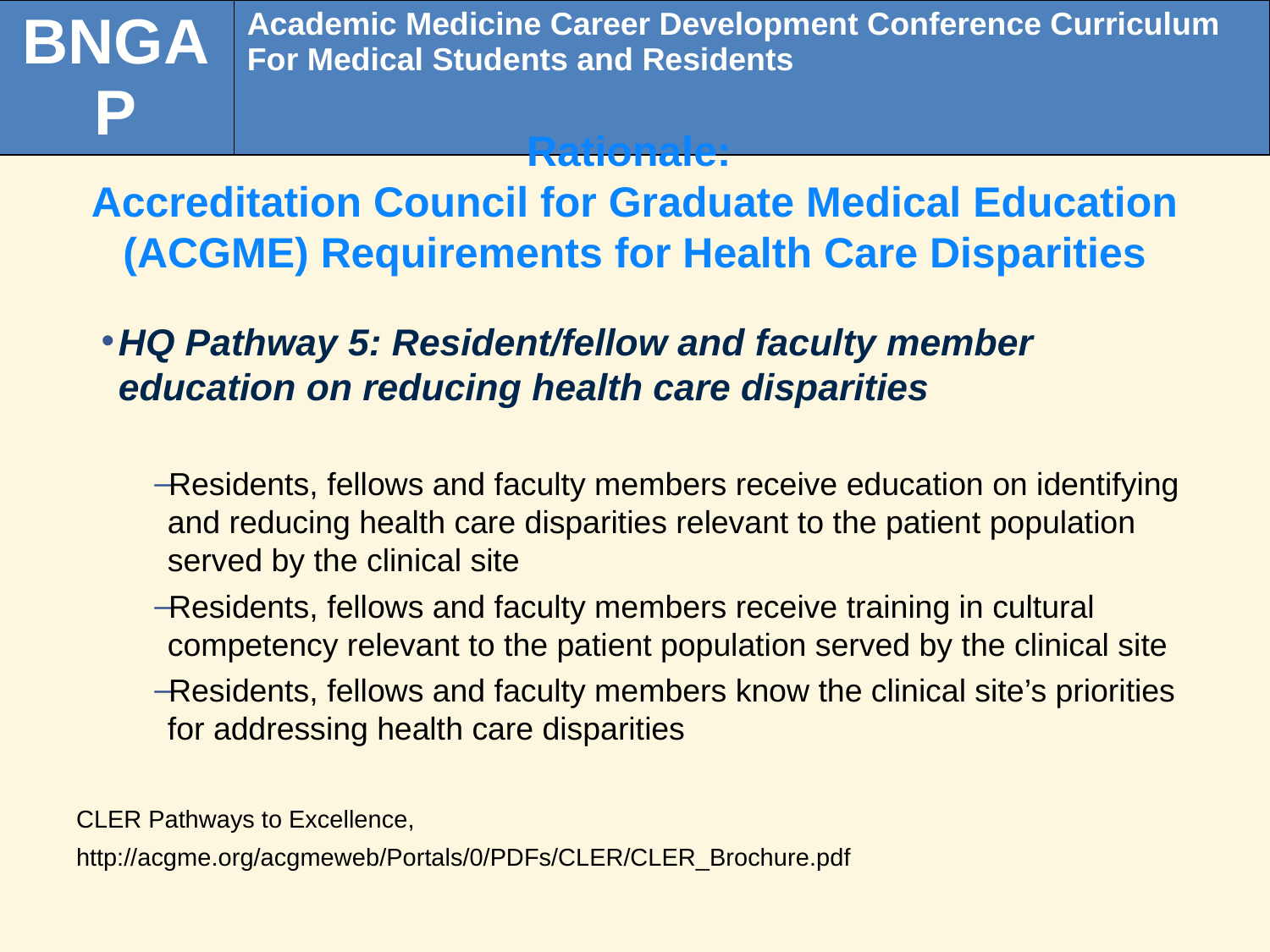

# Rationale: Accreditation Council for Graduate Medical Education (ACGME) Requirements for Health Care Disparities
HQ Pathway 5: Resident/fellow and faculty member education on reducing health care disparities
Residents, fellows and faculty members receive education on identifying and reducing health care disparities relevant to the patient population served by the clinical site
Residents, fellows and faculty members receive training in cultural competency relevant to the patient population served by the clinical site
Residents, fellows and faculty members know the clinical site’s priorities for addressing health care disparities
CLER Pathways to Excellence,
http://acgme.org/acgmeweb/Portals/0/PDFs/CLER/CLER_Brochure.pdf

## Slide 17
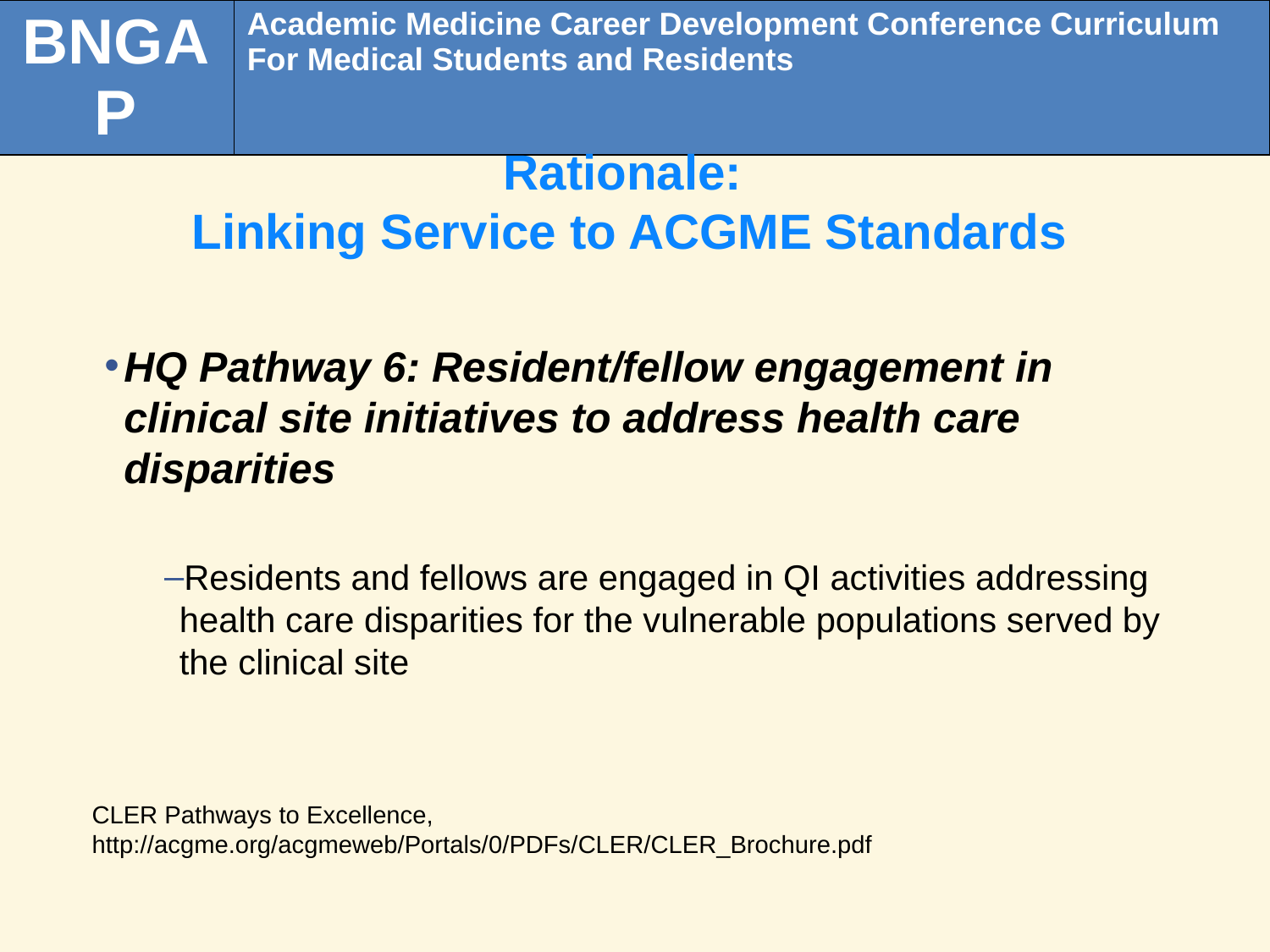

# Rationale: Linking Service to ACGME Standards
HQ Pathway 6: Resident/fellow engagement in clinical site initiatives to address health care disparities
Residents and fellows are engaged in QI activities addressing health care disparities for the vulnerable populations served by the clinical site
CLER Pathways to Excellence,
http://acgme.org/acgmeweb/Portals/0/PDFs/CLER/CLER_Brochure.pdf

## Slide 18
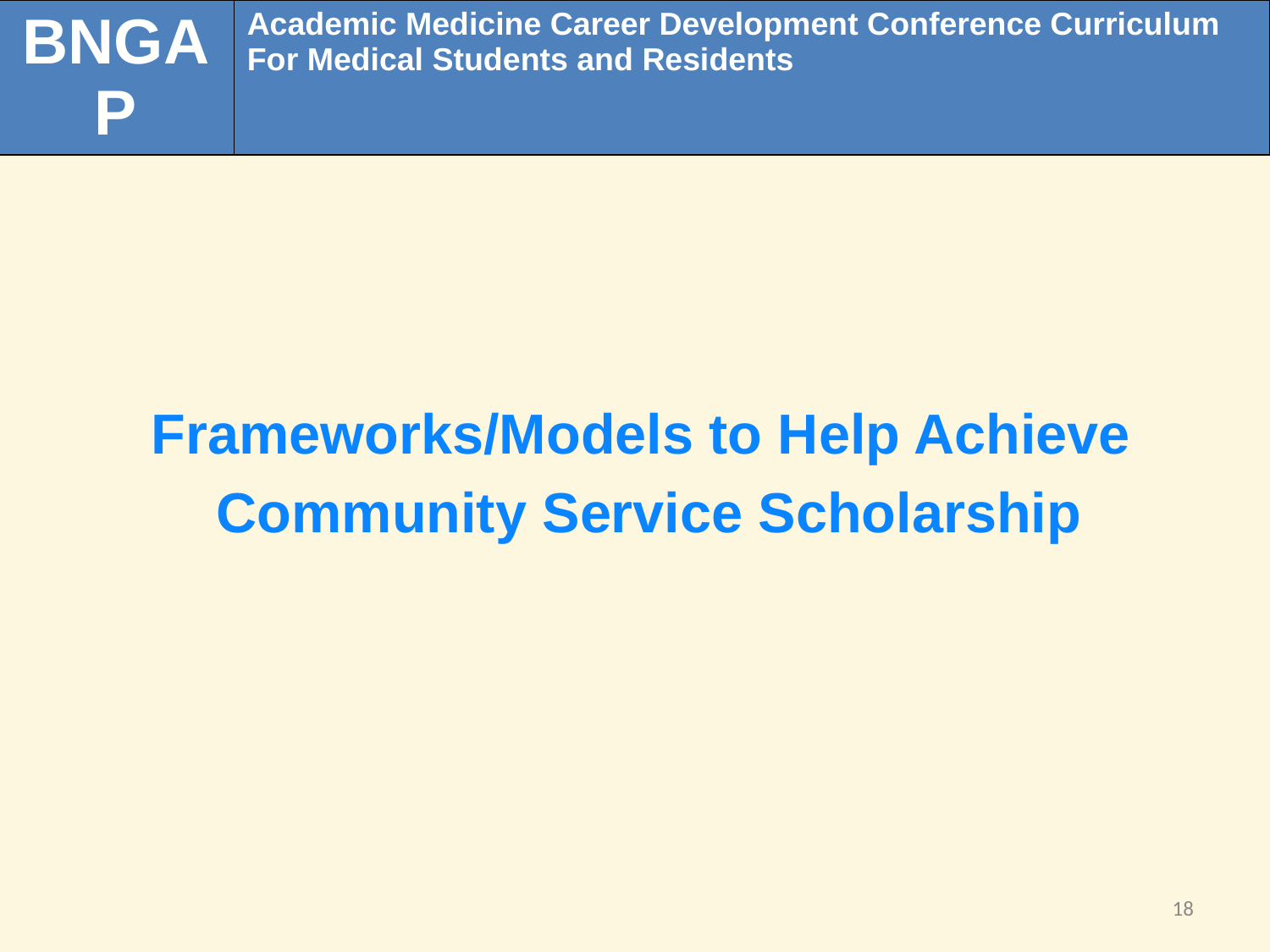

Frameworks/Models to Help Achieve
Community Service Scholarship
18

## Slide 19
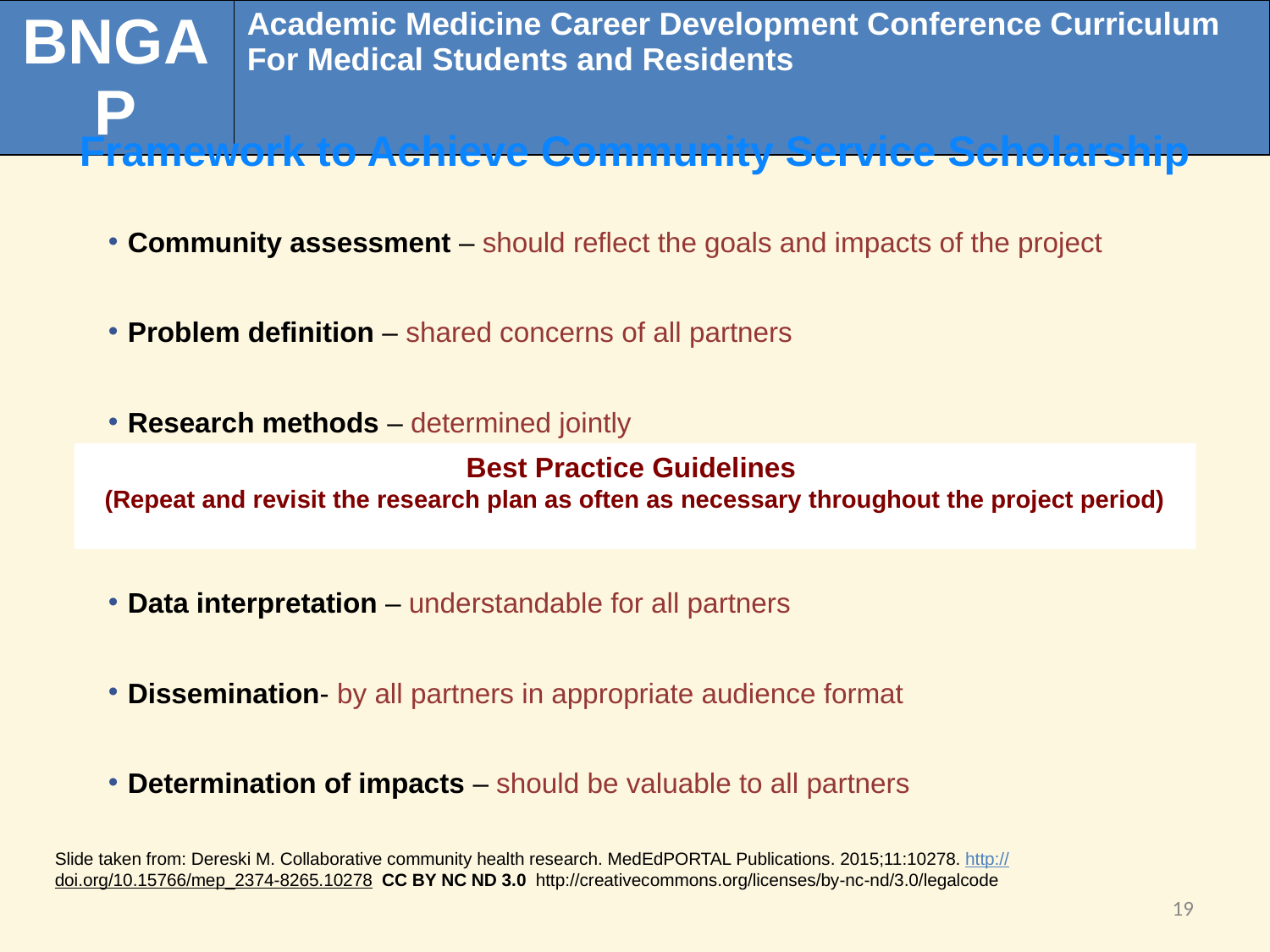

# Framework to Achieve Community Service Scholarship
Community assessment – should reflect the goals and impacts of the project
Problem definition – shared concerns of all partners
Research methods – determined jointly
Data collection and analysis – shared with all partners
Data interpretation – understandable for all partners
Dissemination- by all partners in appropriate audience format
Determination of impacts – should be valuable to all partners
Best Practice Guidelines
(Repeat and revisit the research plan as often as necessary throughout the project period)
Slide taken from: Dereski M. Collaborative community health research. MedEdPORTAL Publications. 2015;11:10278. http://doi.org/10.15766/mep_2374-8265.10278 CC BY NC ND 3.0 http://creativecommons.org/licenses/by-nc-nd/3.0/legalcode
19

## Slide 20
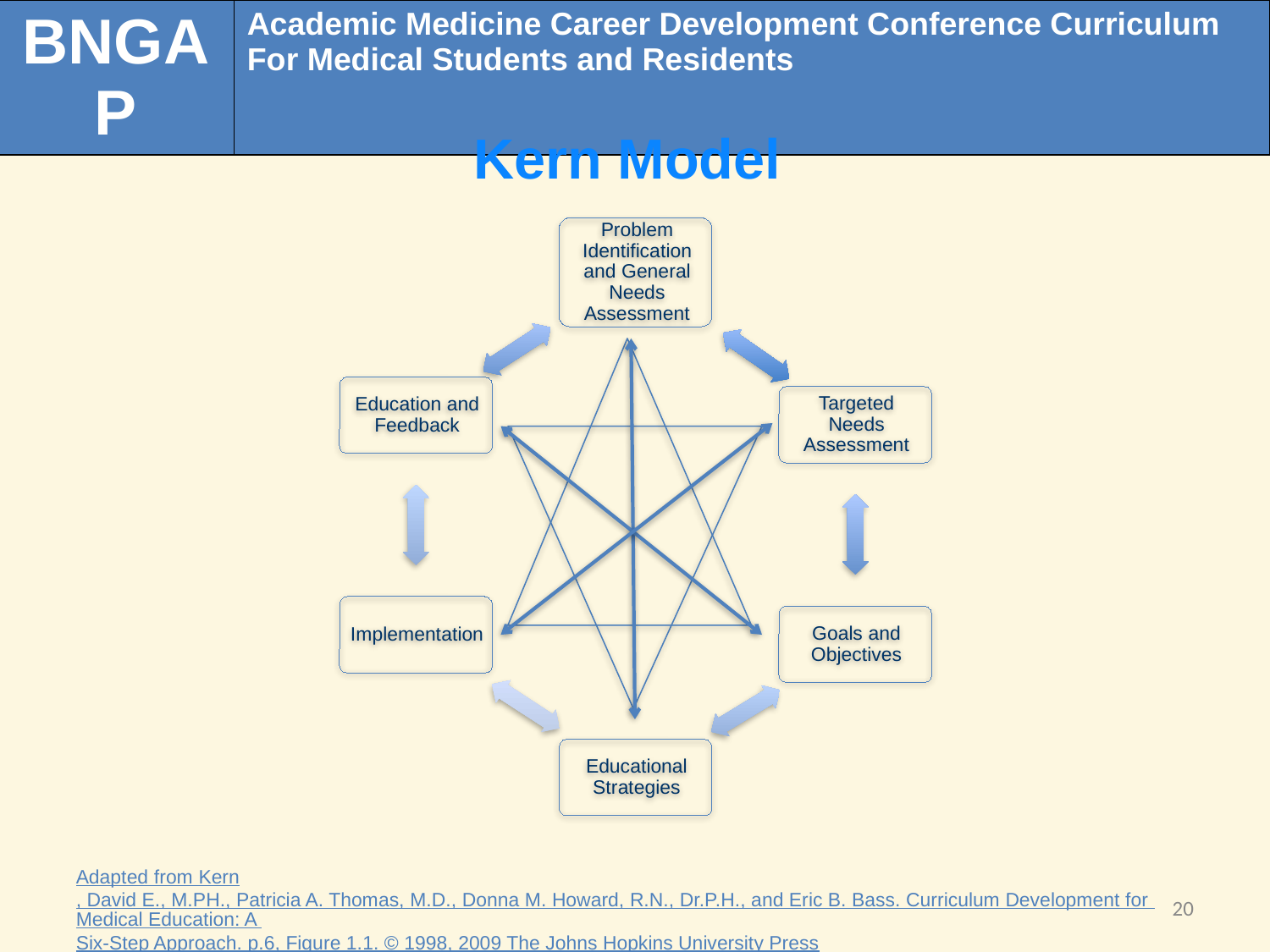

Kern Model
Adapted from Kern, David E., M.PH., Patricia A. Thomas, M.D., Donna M. Howard, R.N., Dr.P.H., and Eric B. Bass. Curriculum Development for Medical Education: A Six-Step Approach. p.6, Figure 1.1. © 1998, 2009 The Johns Hopkins University Press
20

## Slide 21
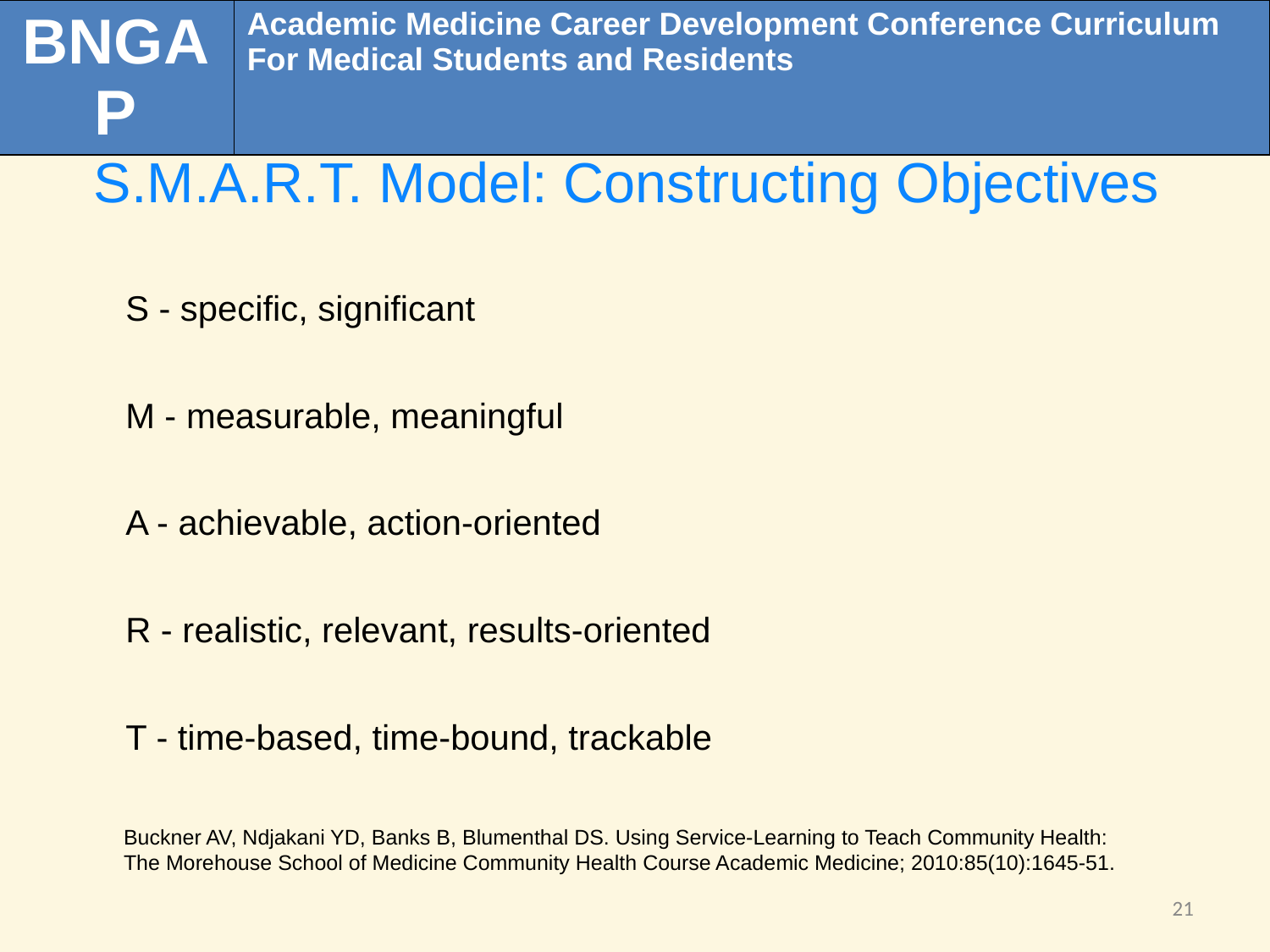

# S.M.A.R.T. Model: Constructing Objectives
S - specific, significant
M - measurable, meaningful
A - achievable, action-oriented
R - realistic, relevant, results-oriented
T - time-based, time-bound, trackable
Buckner AV, Ndjakani YD, Banks B, Blumenthal DS. Using Service-Learning to Teach Community Health: The Morehouse School of Medicine Community Health Course Academic Medicine; 2010:85(10):1645-51.
21

## Slide 22
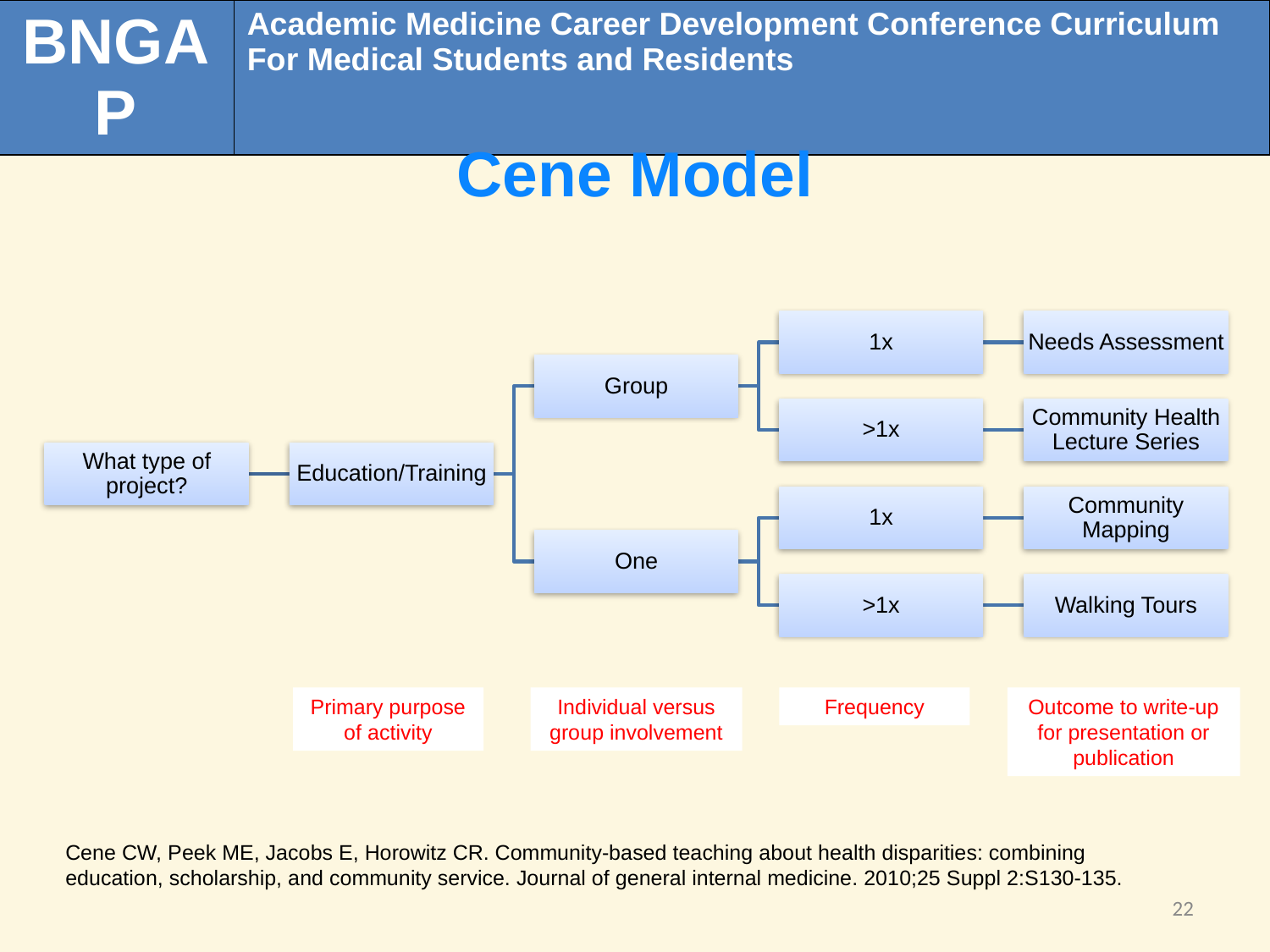

Cene Model
Outcome to write-up for presentation or publication
Primary purpose of activity
Individual versus group involvement
Frequency
Cene CW, Peek ME, Jacobs E, Horowitz CR. Community-based teaching about health disparities: combining education, scholarship, and community service. Journal of general internal medicine. 2010;25 Suppl 2:S130-135.
22

## Slide 23
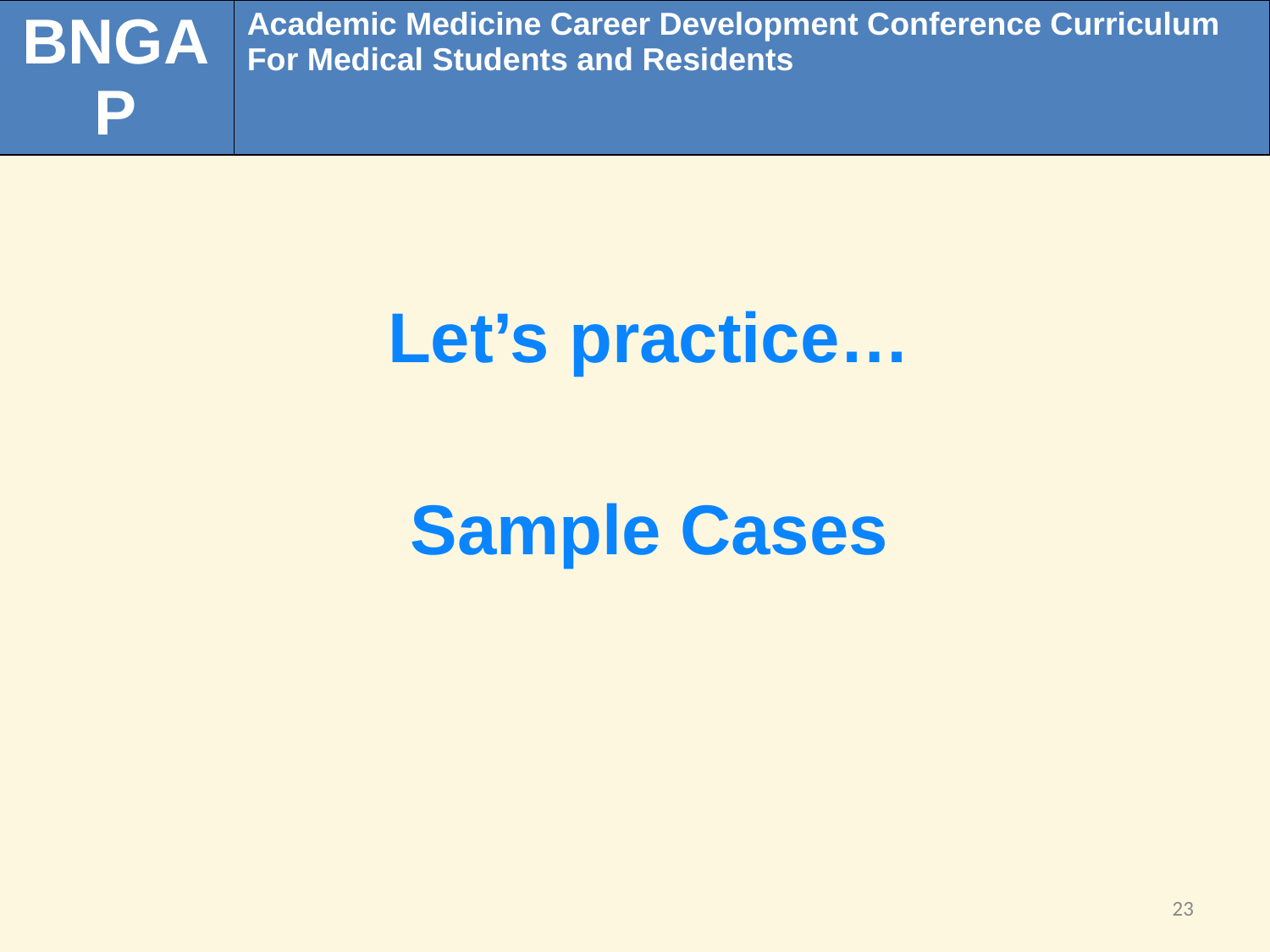

Let’s practice…
Sample Cases
23

## Slide 24
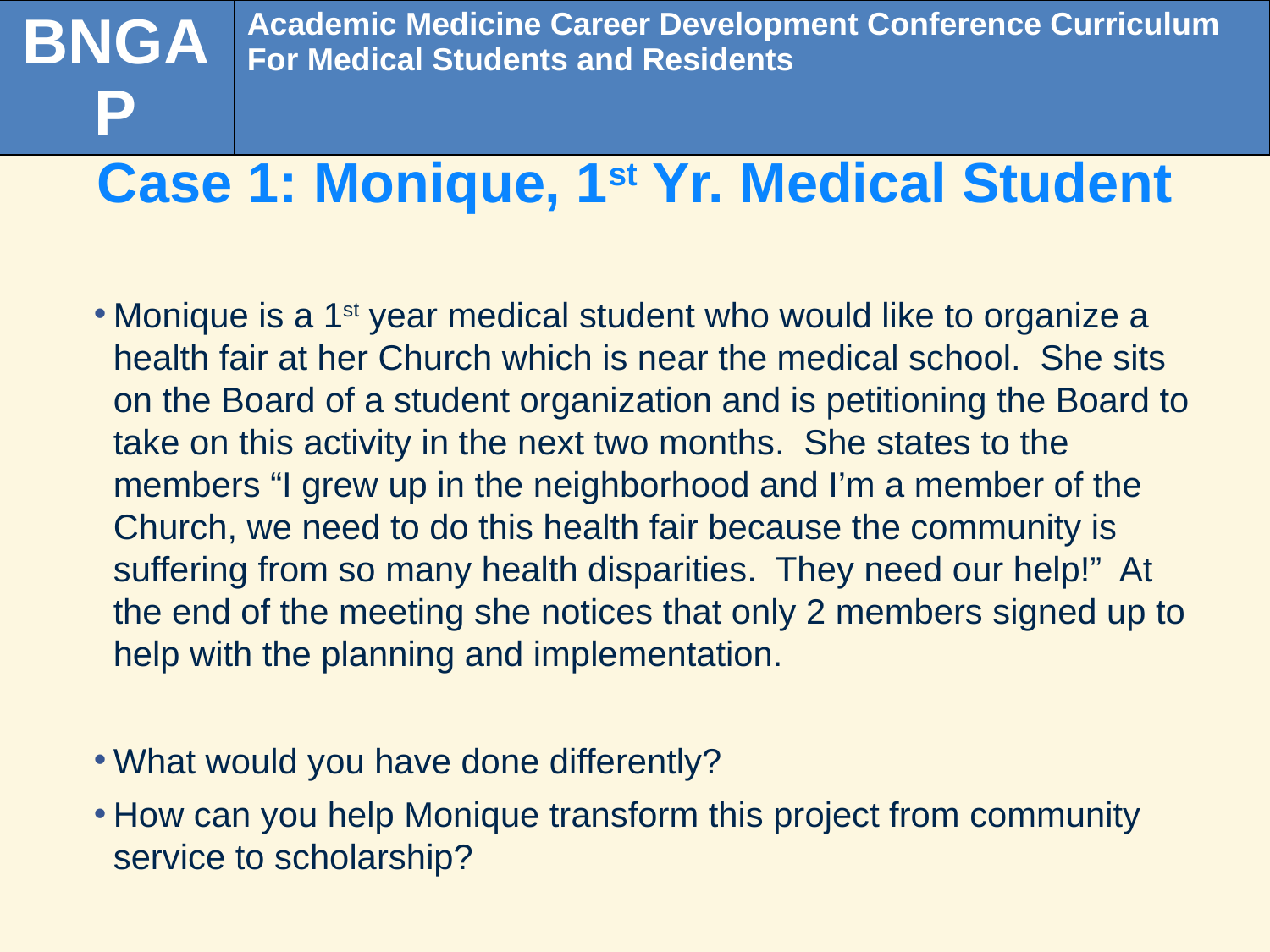

# Case 1: Monique, 1st Yr. Medical Student
Monique is a 1st year medical student who would like to organize a health fair at her Church which is near the medical school. She sits on the Board of a student organization and is petitioning the Board to take on this activity in the next two months. She states to the members “I grew up in the neighborhood and I’m a member of the Church, we need to do this health fair because the community is suffering from so many health disparities. They need our help!” At the end of the meeting she notices that only 2 members signed up to help with the planning and implementation.
What would you have done differently?
How can you help Monique transform this project from community service to scholarship?

## Slide 25
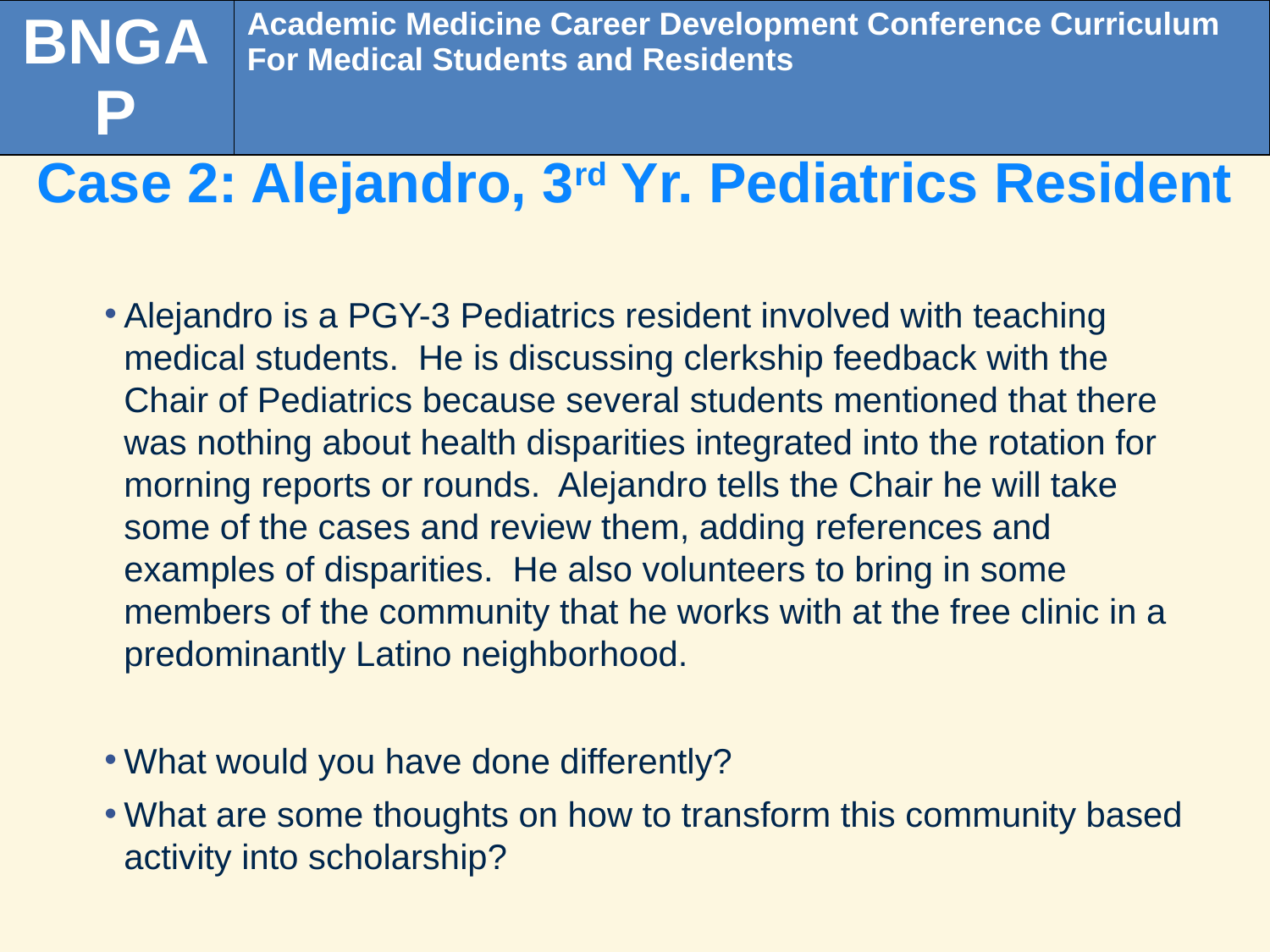

# Case 2: Alejandro, 3rd Yr. Pediatrics Resident
Alejandro is a PGY-3 Pediatrics resident involved with teaching medical students. He is discussing clerkship feedback with the Chair of Pediatrics because several students mentioned that there was nothing about health disparities integrated into the rotation for morning reports or rounds. Alejandro tells the Chair he will take some of the cases and review them, adding references and examples of disparities. He also volunteers to bring in some members of the community that he works with at the free clinic in a predominantly Latino neighborhood.
What would you have done differently?
What are some thoughts on how to transform this community based activity into scholarship?

## Slide 26
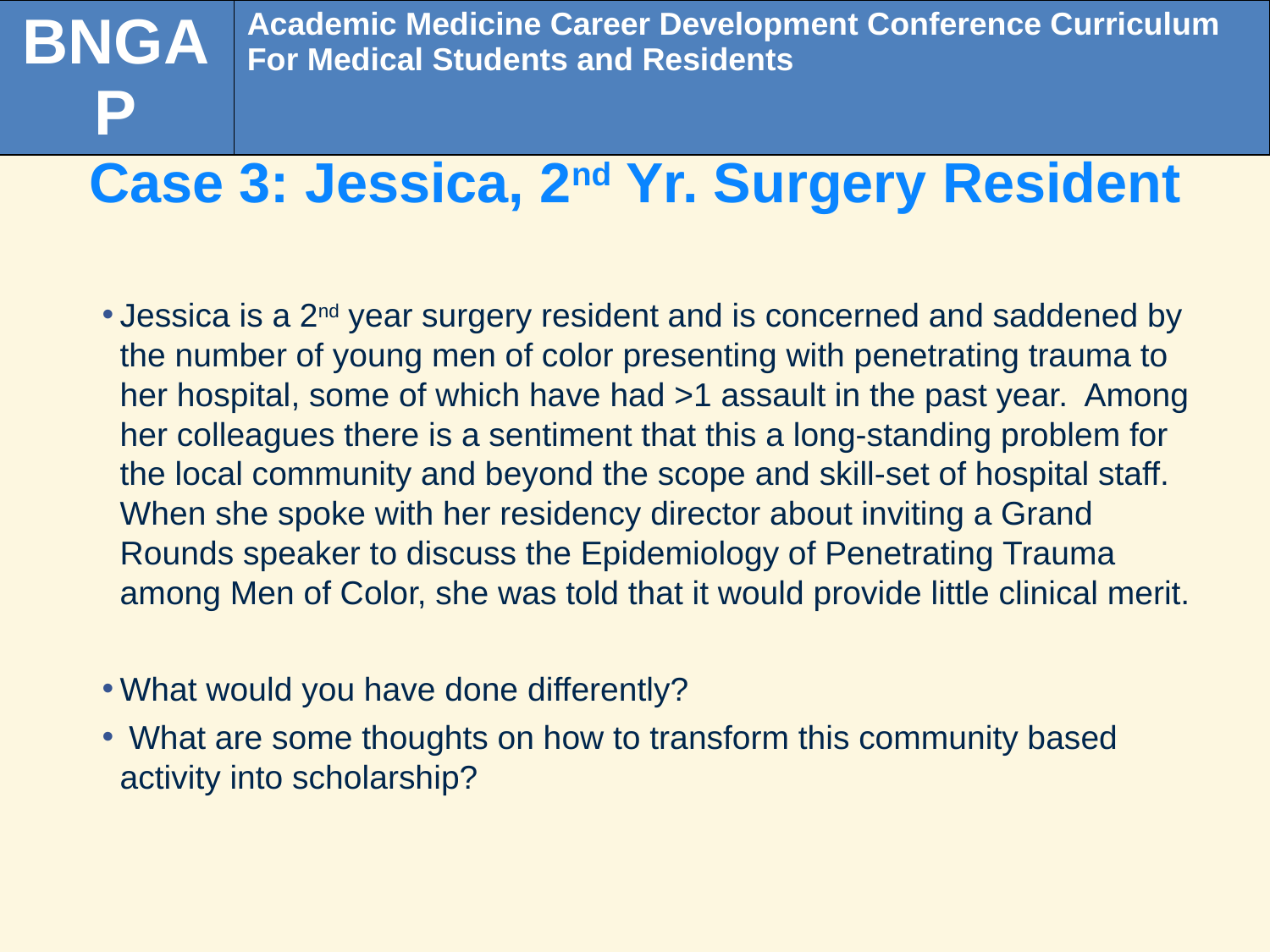

# Case 3: Jessica, 2nd Yr. Surgery Resident
Jessica is a 2nd year surgery resident and is concerned and saddened by the number of young men of color presenting with penetrating trauma to her hospital, some of which have had >1 assault in the past year. Among her colleagues there is a sentiment that this a long-standing problem for the local community and beyond the scope and skill-set of hospital staff. When she spoke with her residency director about inviting a Grand Rounds speaker to discuss the Epidemiology of Penetrating Trauma among Men of Color, she was told that it would provide little clinical merit.
What would you have done differently?
 What are some thoughts on how to transform this community based activity into scholarship?

## Slide 27
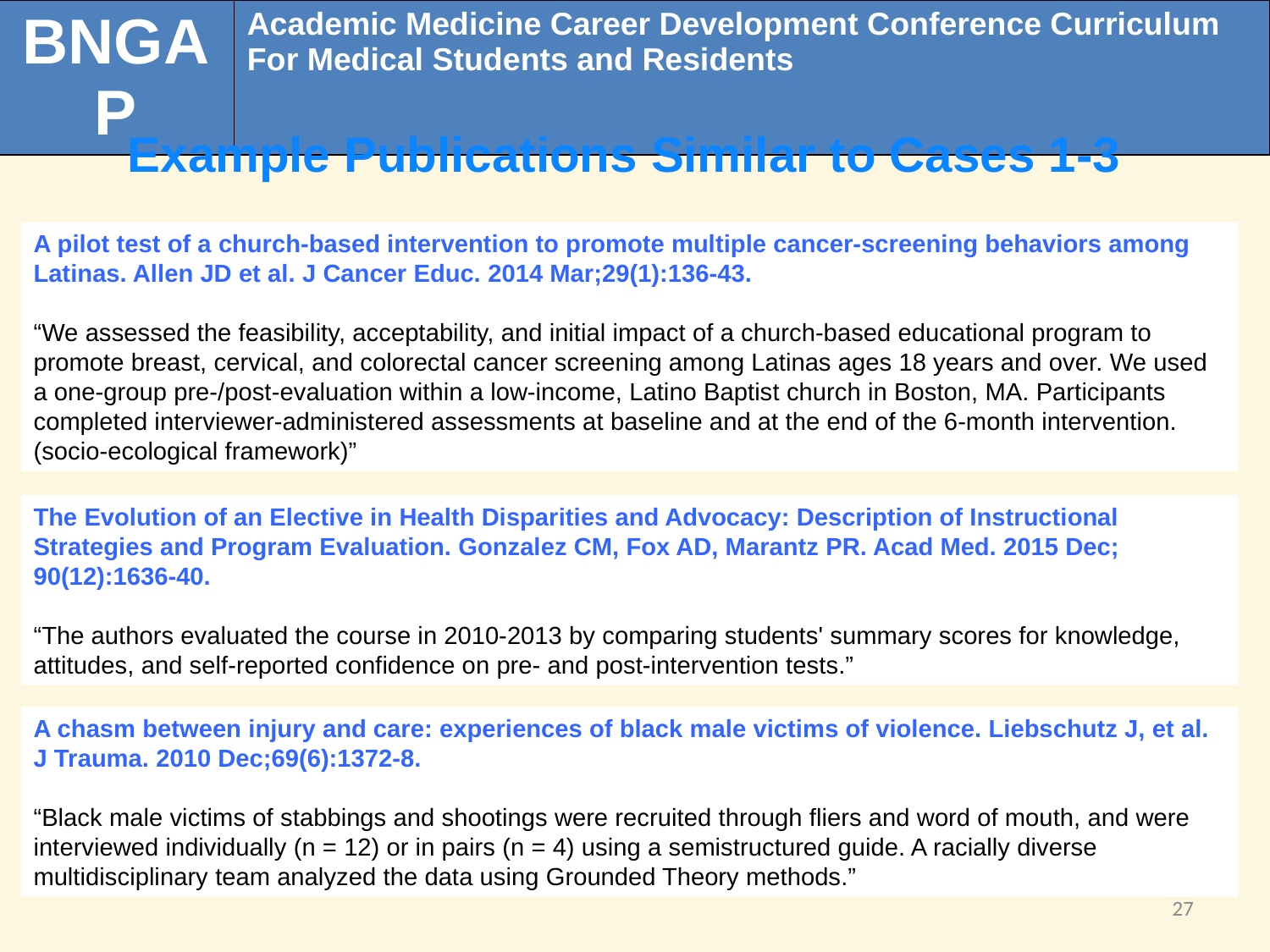

Example Publications Similar to Cases 1-3
A pilot test of a church-based intervention to promote multiple cancer-screening behaviors among Latinas. Allen JD et al. J Cancer Educ. 2014 Mar;29(1):136-43.
“We assessed the feasibility, acceptability, and initial impact of a church-based educational program to promote breast, cervical, and colorectal cancer screening among Latinas ages 18 years and over. We used a one-group pre-/post-evaluation within a low-income, Latino Baptist church in Boston, MA. Participants completed interviewer-administered assessments at baseline and at the end of the 6-month intervention. (socio-ecological framework)”
The Evolution of an Elective in Health Disparities and Advocacy: Description of Instructional Strategies and Program Evaluation. Gonzalez CM, Fox AD, Marantz PR. Acad Med. 2015 Dec; 90(12):1636-40.
“The authors evaluated the course in 2010-2013 by comparing students' summary scores for knowledge, attitudes, and self-reported confidence on pre- and post-intervention tests.”
A chasm between injury and care: experiences of black male victims of violence. Liebschutz J, et al. J Trauma. 2010 Dec;69(6):1372-8.
“Black male victims of stabbings and shootings were recruited through fliers and word of mouth, and were interviewed individually (n = 12) or in pairs (n = 4) using a semistructured guide. A racially diverse multidisciplinary team analyzed the data using Grounded Theory methods.”
27

## Slide 28
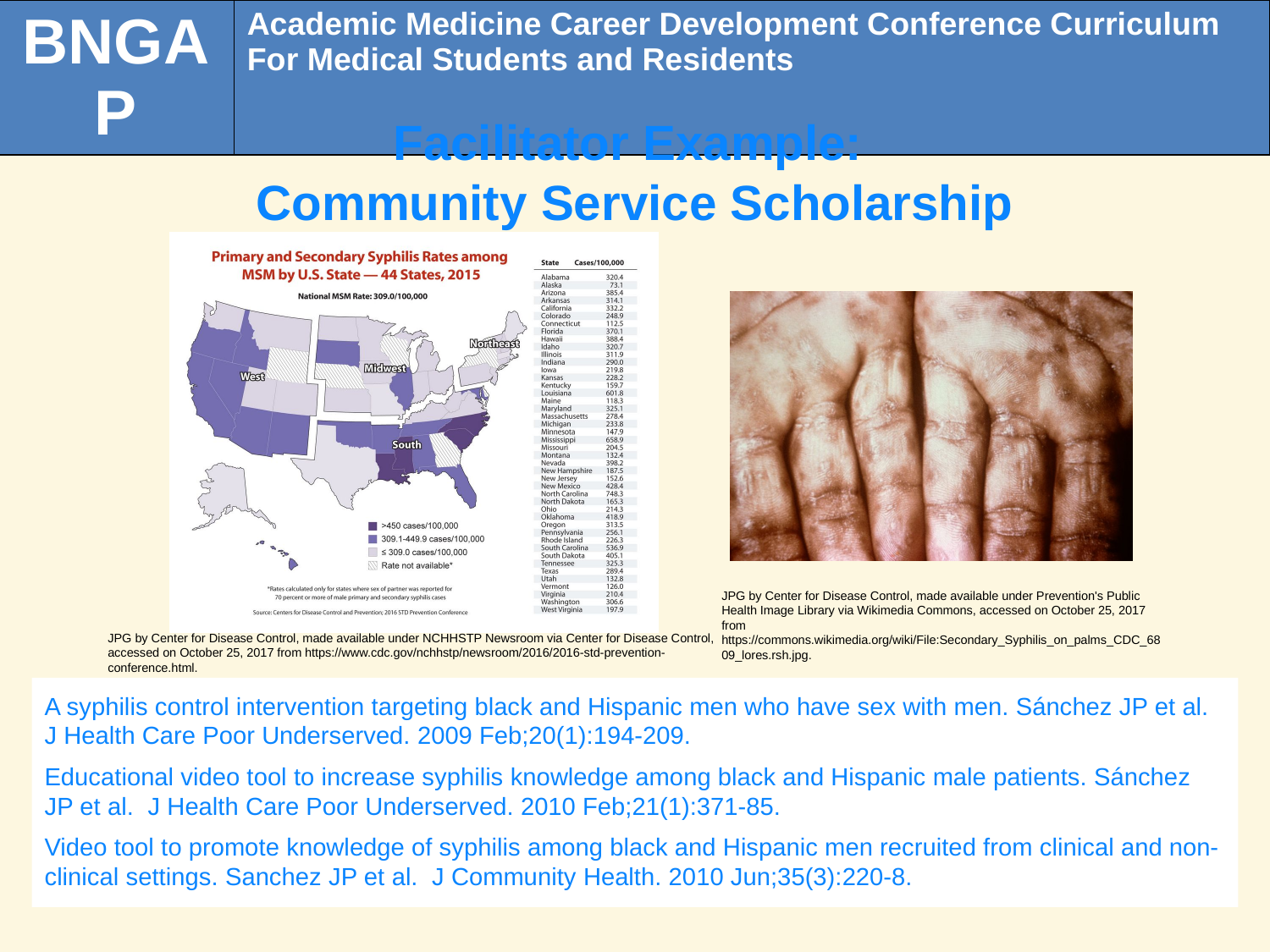

# Facilitator Example: Community Service Scholarship
JPG by Center for Disease Control, made available under Prevention's Public Health Image Library via Wikimedia Commons, accessed on October 25, 2017 from https://commons.wikimedia.org/wiki/File:Secondary_Syphilis_on_palms_CDC_6809_lores.rsh.jpg.
JPG by Center for Disease Control, made available under NCHHSTP Newsroom via Center for Disease Control, accessed on October 25, 2017 from https://www.cdc.gov/nchhstp/newsroom/2016/2016-std-prevention-conference.html.
A syphilis control intervention targeting black and Hispanic men who have sex with men. Sánchez JP et al. J Health Care Poor Underserved. 2009 Feb;20(1):194-209.
Educational video tool to increase syphilis knowledge among black and Hispanic male patients. Sánchez JP et al. J Health Care Poor Underserved. 2010 Feb;21(1):371-85.
Video tool to promote knowledge of syphilis among black and Hispanic men recruited from clinical and non-clinical settings. Sanchez JP et al. J Community Health. 2010 Jun;35(3):220-8.

## Slide 29
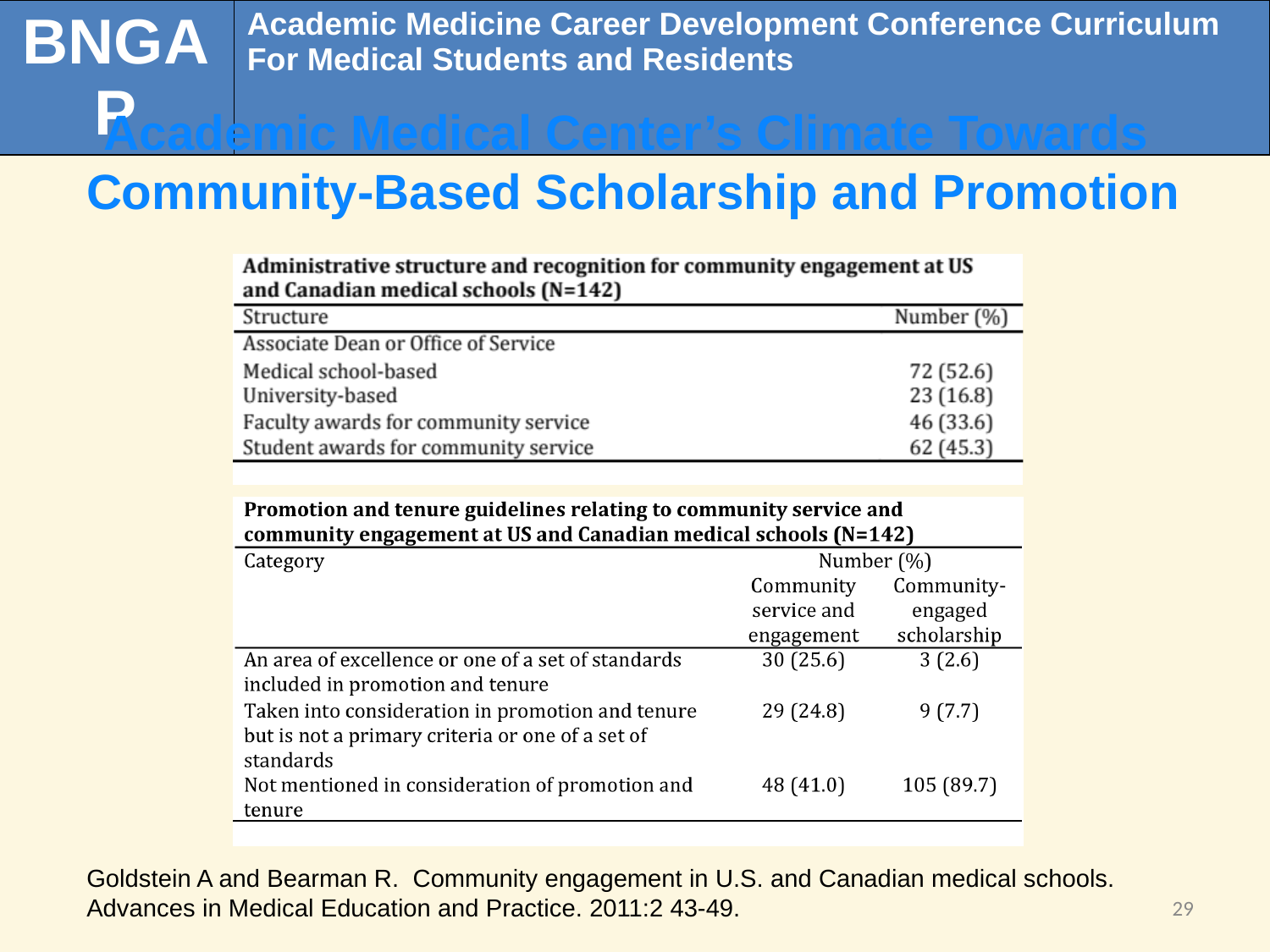

# Academic Medical Center’s Climate Towards Community-Based Scholarship and Promotion
Goldstein A and Bearman R. Community engagement in U.S. and Canadian medical schools. Advances in Medical Education and Practice. 2011:2 43-49.
29

## Slide 30
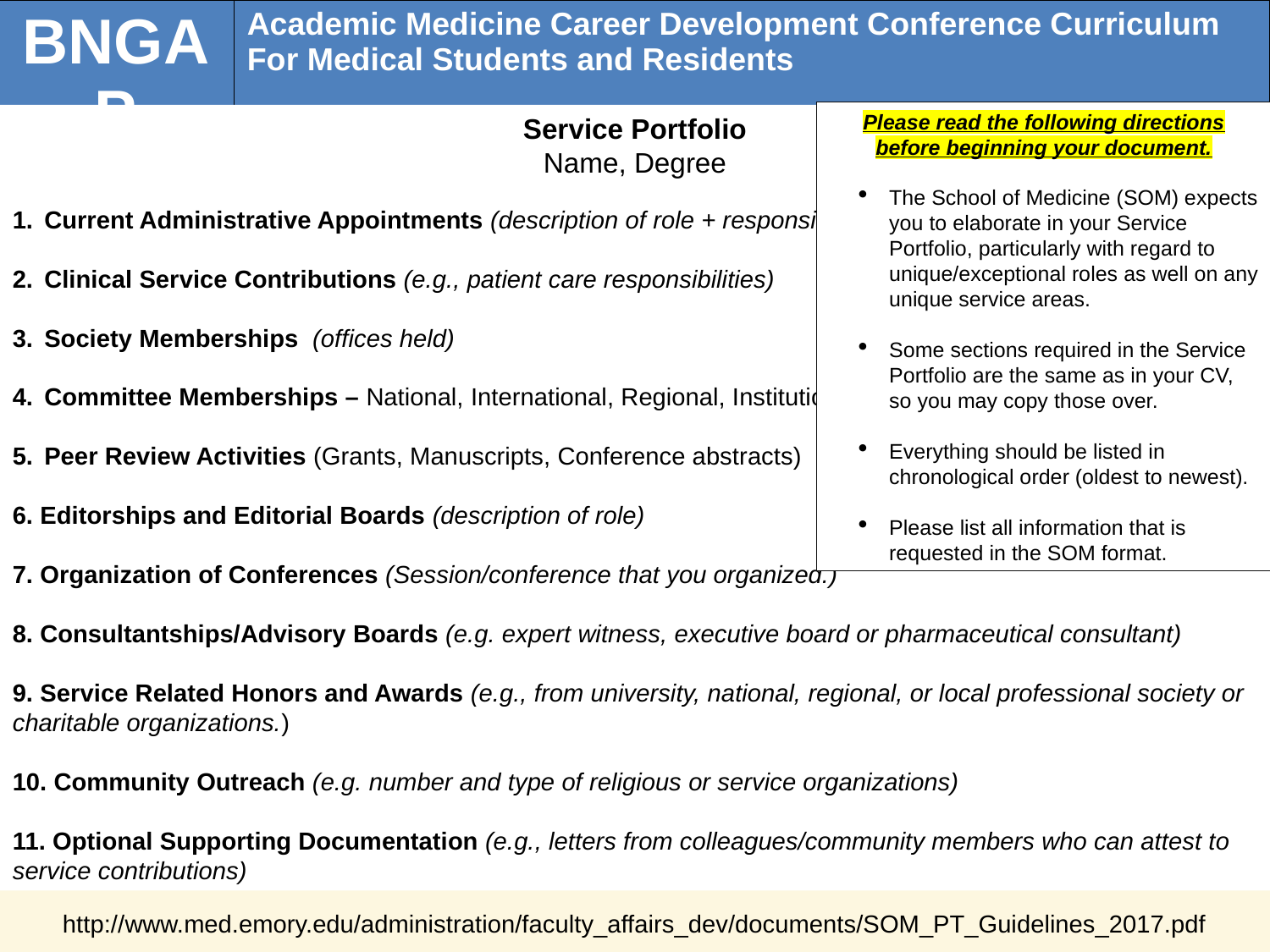

Please read the following directions before beginning your document.
The School of Medicine (SOM) expects you to elaborate in your Service Portfolio, particularly with regard to unique/exceptional roles as well on any unique service areas.
Some sections required in the Service Portfolio are the same as in your CV, so you may copy those over.
Everything should be listed in chronological order (oldest to newest).
Please list all information that is requested in the SOM format.
Service Portfolio
Name, Degree
Current Administrative Appointments (description of role + responsibilities)
Clinical Service Contributions (e.g., patient care responsibilities)
Society Memberships (offices held)
Committee Memberships – National, International, Regional, Institutional
Peer Review Activities (Grants, Manuscripts, Conference abstracts)
6. Editorships and Editorial Boards (description of role)
7. Organization of Conferences (Session/conference that you organized.)
8. Consultantships/Advisory Boards (e.g. expert witness, executive board or pharmaceutical consultant)
9. Service Related Honors and Awards (e.g., from university, national, regional, or local professional society or charitable organizations.)
10. Community Outreach (e.g. number and type of religious or service organizations)
11. Optional Supporting Documentation (e.g., letters from colleagues/community members who can attest to service contributions)
http://www.med.emory.edu/administration/faculty_affairs_dev/documents/SOM_PT_Guidelines_2017.pdf

## Slide 31
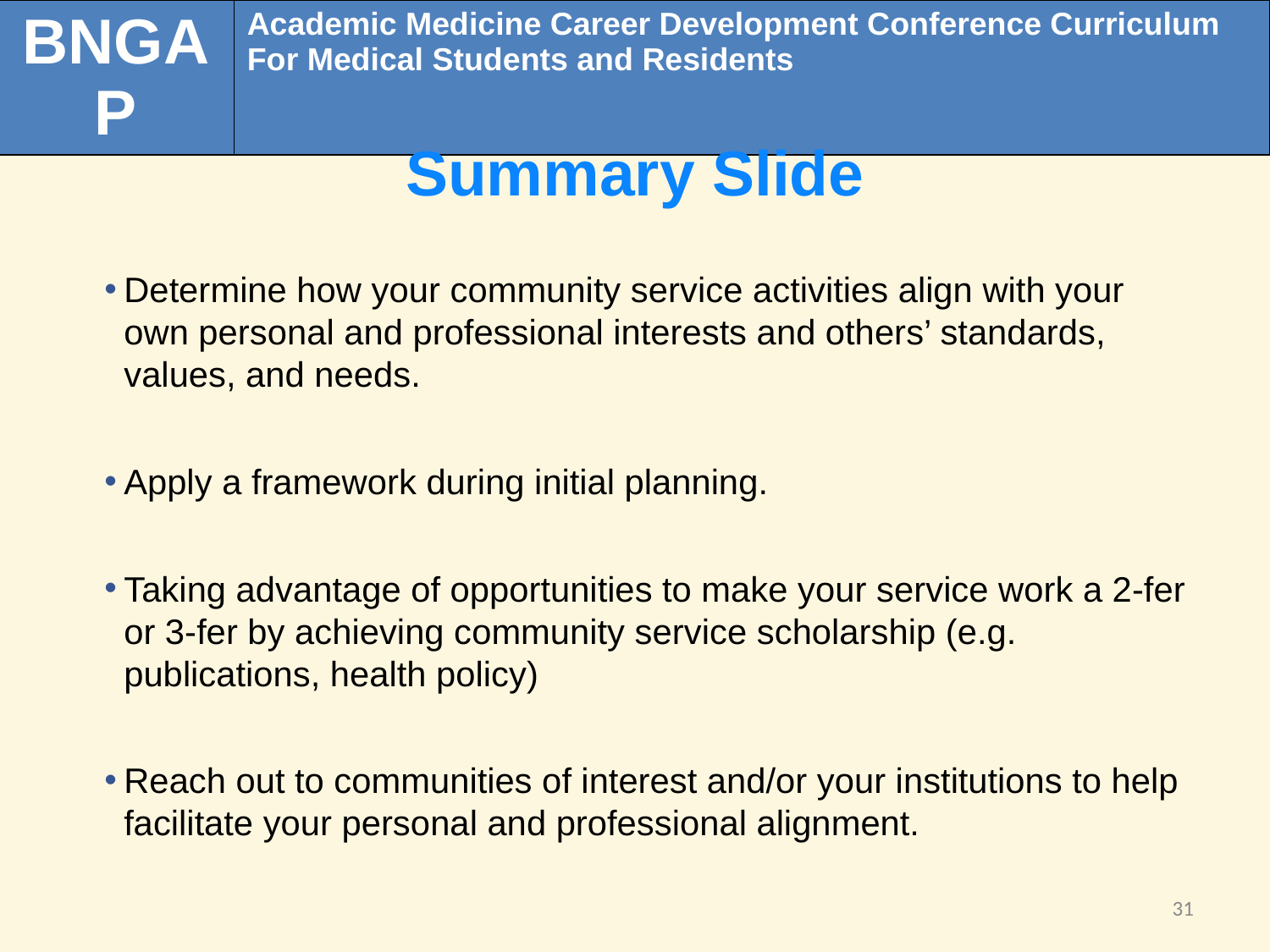

# Summary Slide
Determine how your community service activities align with your own personal and professional interests and others’ standards, values, and needs.
Apply a framework during initial planning.
Taking advantage of opportunities to make your service work a 2-fer or 3-fer by achieving community service scholarship (e.g. publications, health policy)
Reach out to communities of interest and/or your institutions to help facilitate your personal and professional alignment.
31

## Slide 32
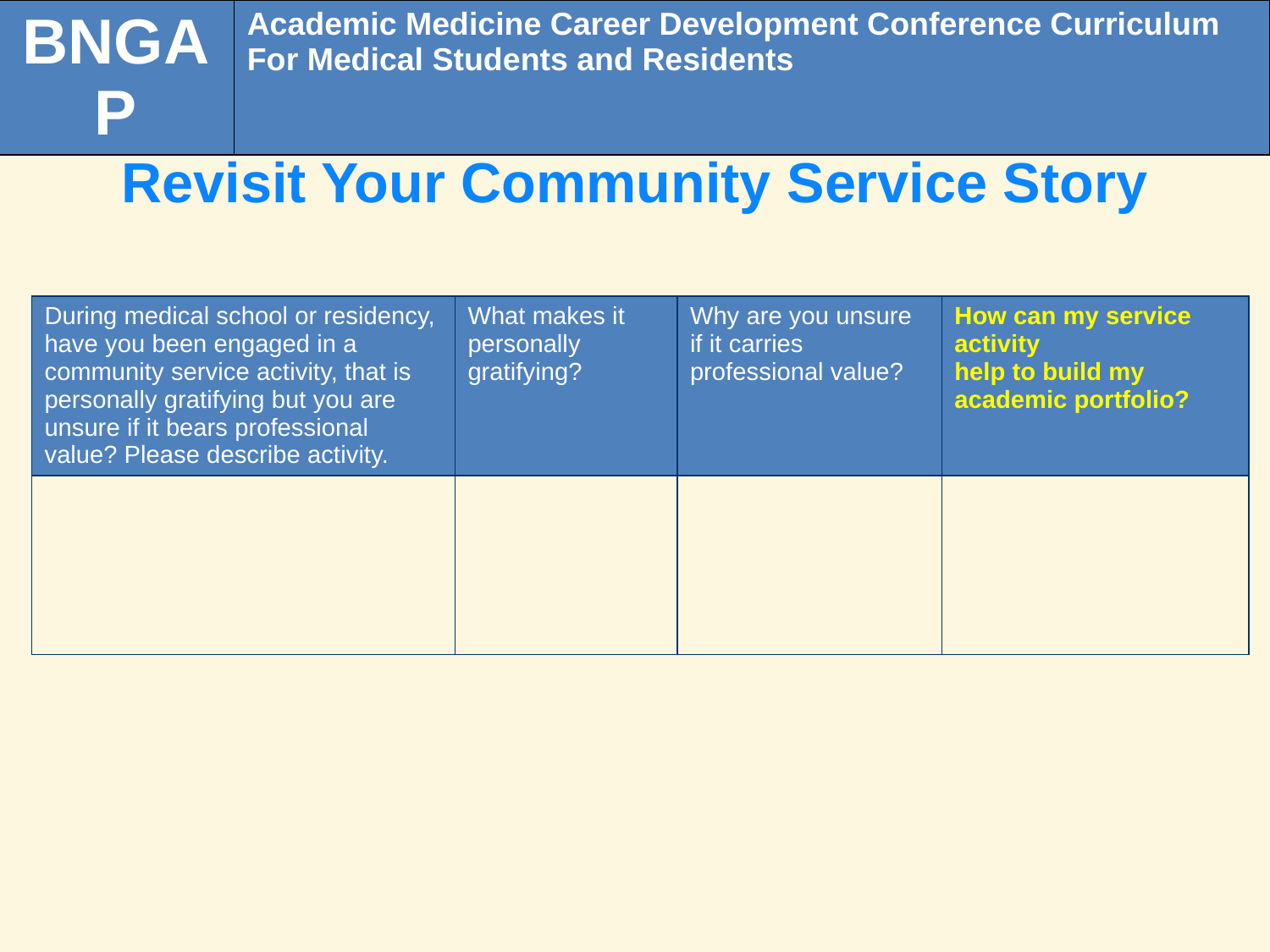

# Revisit Your Community Service Story
| During medical school or residency, have you been engaged in a community service activity, that is personally gratifying but you are unsure if it bears professional value? Please describe activity. | What makes it personally gratifying? | Why are you unsure if it carries professional value? | How can my service activity help to build my academic portfolio? |
| --- | --- | --- | --- |
| | | | |

## Slide 33
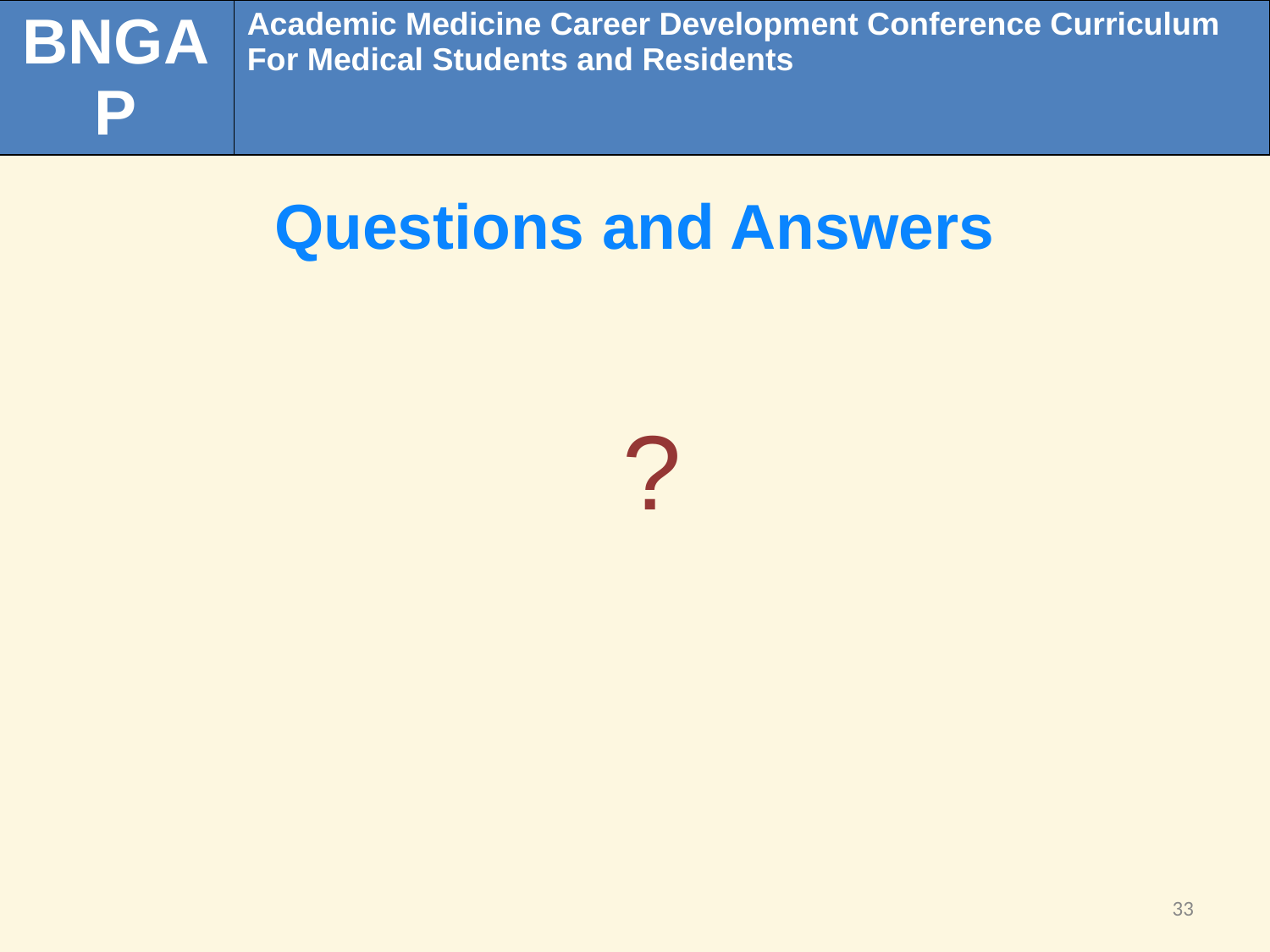

# Questions and Answers
?
33
