# Supplementary material for: Helping Trainees Develop Scholarship in Academic Medicine From Community Service
Source: MedEdPORTAL. 2017 Dec 14;13:10659. doi: 10.15766/mep_2374-8265.10659 (PMC6338162; doi:10.15766/mep_2374-8265.10659)
Supplement: Supplementary file 1 — A. PowerPoint Presentation.pptx B. Slide Instructions.docx C. Train the Trainer Workshop Video.mp4 D. Worksheet.doc E. Case Scenarios.doc F. Evaluation Form.doc [file mep-13-10659-s001.zip › B. Slide Instructions.docx]

**Helping Trainees Develop Scholarship in Academic Medicine from Community Service**

**Facilitator Instructional Guide**

**Overall Goals**

The goals of our workshop are to provide insight on the benefits of completing community service scholarship, especially in relation to an academic medicine career, and how to transform community service work to scholarship. This workshop may have 1-2 facilitators.

**Workshop Objectives**

1. Define the terms community service, community engagement, and service learning

2. Describe frameworks to help achieve community service scholarship

3. Apply steps to achieving community service scholarship: sample cases and personal stories

**Workshop Handouts and Materials**

1. BNGAP Service Scholarship Worksheet: Your Personal Story
   1. This can be handed out at the beginning for participants’ to reflect on their historical or current community service activities

2. BNGAP Service Scholarship Case Scenarios

3. BNGAP Service Scholarship Evaluation Forms

**Suggested Agenda and Timeline**

- Slide 1-22: 25 minutes
- Slides 23-27 (Group Exercise and discussion): 15 minutes
- Slide 28-32: 15 minutes
- Questions and answers: 5 minutes

**Slide Instructions**

**Slide 1:**

Title slide

**Slide 2**:

The facilitators should introduce themselves to the audience and discuss their roles in their respective institutions. At the point the audience should be informed that we will be covering a lot of topics and some theories and frameworks and it is okay if they don’t get everything. Our goal is to raise their awareness and give them insight into this landscape.

**Slide 3:**

Facilitator should state objectives and time line for the workshop and emphasize that this workshop will give an overview of building community service scholarship.

**Slide 4 – Your Community Service Story Handout:**

This handout provides an opportunity for the trainees to reflect on a community service activity that they find to be personally gratifying but are unsure if it carries professional value. Students should be allotted 3-5 minutes to complete exercise.

Prior to the implementation of the workshop, the facilitator should provide their own community service story by filling out the second row (the last author has provided an example).

Ask two to three individuals to share their responses. One or both of the speakers may be willing to share their own responses to the question if participants are unable to come up with examples.

Examples of professional value can be increased opportunities for awards, to meet institutional service requirements, improve chances of matching into a specific residency, publishing, receiving grant-funding, etc.

Remind participants that this workshop will help participants think about how to better align their community service activities with their professional interests and potentially transform their activities into forms of scholarship that can serve as a foundation for an academic portfolio.

**Slide 5:**

Often the terms community service, community engagement, and service learning are used interchangeably yet they are distinct. Here we review definitions for clarity.

- Community Service
  - Services **volunteered** by individuals or an organization **to benefit a community** **or its institutions.**
- Community Engagement
  - The process of **working collaboratively with and through groups** of people affiliated by geographic proximity, special interest, or similar situations to address issues affecting the well-being of those people.
- Service Learning
  - A form of community-centered experiential education that places emerging health professionals in community-generated service projects and **provides structured opportunities for reflection on the broader social, economic, and political contexts of health.^1^**

Whereas many individuals participate in community service, community engagement (such as community engaged research or teaching) is structured to ensure that those being helped are involved in the design, implementation and evaluation of any activity.

Service learning the active process of ensuring that students involved in a community service project have structured reflection that allows for a broader understanding of the social, economic and political contexts faced by a community.

Photo 1

https://flic.kr/p/Rax1

CC BY-NC-ND-2.0

https://creativecommons.org/licenses/by-nc-nd/2.0/

Photo 2

By Bonvallite - Own work, CC BY-SA 3.0, https://commons.wikimedia.org/w/index.php?curid=23522959

Image 3 (Service Learning Diagram)

Created by Co-Author J.P. Sanchez MD, MPH

**Slide 6:**

You should be aware that you could serve a broad array of communities.

You can serve local communities (e.g. schools, civic organizations) or remote communities.

**Slide 7:**

You can serve your academic community as admission tour guide or interviewer.

This would also qualify as academic citizenship, which focuses on the roles and responsibilities of trainees and faculty in serving their academic community.

Good academic citizenship by students can be recognized through awards or can facilitate faculty promotion.^2^

**Slide 8:**

Community service scholarship is a different term. It involves the process of structuring and documenting your community service work in a scholarly fashion. Products of scholarly work can be peer-reviewed publications, grant funding to continue your work, awards in recognition of your work, and the passage of policies.

Scholarly products will make you a more competitive applicant for residency, fellowship and for a future academic career and appointment.

**Slide 9:**

You are better able to achieve community service scholarship when you consider value based alignment.

How does your community service activity align with your…

- Personal passion
- Professional interests
- Institutional, school, or departmental mission/vision/objectives
- Network/community specific needs or disparities

Reflecting on these questions can help you to better articulate how your community service activity benefits your personal and professional growth, the community being served or the interest of your academic community.

In addition, such an approach can help you to strategically allocate your efforts to address a defined community service void or gap. Defined voids and gaps are more likely to lead to a scholarly product.

**Slide 10:**

By reflecting on the value of your activities for yourself, for the community being served and for your institution you can leverage your chances of having the activity enhance your professional development, portfolio and promotion. Your community service work can lead to two-fers and three-fers by leading to:

- Improved skill sets: management, organizational, communication, leadership, etc.
- Elective credit for your efforts
- Recognition inside and outside your institution (awards, certificates, scholarships, leadership opportunities)
- Peer review publications: curriculum submitted to AAMC MedEdPORTAL, journals, book chapters, commentaries
- Presentations: oral or posters at regional or national conferences, committee reports, policy manuals, etc.

**Slide 11:**

Did you know that the majority of you and your colleagues have been involved in community service very early in your professional journey.

Of those who took the MCAT in 2015, 84.0% volunteered in the healthcare field and nearly the same amount 76.0% volunteered in the non-healthcare field. Volunteering was reported just as much as shadowing a physician or other healthcare professional. These volunteer experiences not only helped you to be a more competitive applicant to medical school but can also help you in building a portfolio for an academic medicine career.^3^

**Slide 12:**

As reported by medical student graduates on the 2015 AAMC Graduation Questionnaire, the majority of your colleagues continue to engage in a number of community service activities during medical school. For example, they reported engaging in health education and in pipeline programs for elementary, high school or college students. They engaged in experiences that afforded greater exposure to health disparities, cultural awareness and competence.^4^

And although nearly 2/3 participated in a research project with a faculty member, of which many achieved a peer-reviewed presentation or publication only a quarter reported engaging in a community-based research project. We are here today to raise your awareness on the relevance of and how to transform your community service work into a research or scholarly product.

**Slide 13:**

Here is an example of a void or gap related to community service and education. Despite your level of involvement in the community, surprisingly 31.6% of medical student respondents to the 2014 Graduation Questionnaire reported that there instruction in the role of community health and social service agencies was INADEQUATE; this proportion was higher than for instruction on Public health (27.1%), Health determinants (12.3%), Culturally appropriate care for diverse populations (12.9%), Disease prevention (8.6%), Women’s health (7.6%).^5^

This inadequacy deserves greater attention and presents a “void or gap” in medical or resident education.

**Slide 14:**

Let’s move from what you and your colleagues have documented as your experiences in relation to community service to how medical schools and residency programs are expected to support your community service experiences.

The Liaison Committee on Medical Education (LCME) accreditation standard 6.6 Service Learning is an important standard because it mandates medical school faculty to support medical student participation in service-learning and community service activities.^6^

**Slide 15:**

Another example of a service activity mandated by the LCME is Standard 3.3 which focuses on Diversity/Pipeline programs and Partnerships. By serving your institution in meeting this standard you are helping your school remain accredited and are serving as a good academic citizen.

As many of you work on diversity efforts, remember, through the value based model, you are not only fulfilling your own personal and professional interests but also serving your institution and the surrounding communities. Documenting your efforts and effectiveness also serves the institution in meeting their accreditation standards.

**Slides 16:**

Recently, the Accreditation Council for Graduate Medical Education (ACGME) released the Clinical Learning Environment Review (CLER) a set of standards designed to improve how clinical sites engage resident and fellow physicians in learning to provide safe, high quality patient care. The following 2 pathways listed in the publication highlight the opportunity for residents and fellows to participate in community engaged service activities that can also lead to scholarship.

***HQ Pathway 5: Resident/fellow and faculty member education on reducing health care disparities^7^***

- Formal educational activities that create a shared mental model with regard to health care quality related goals, tools, and techniques are necessary for health care professionals to consistently work in a well-coordinated manner to achieve a true patient-centered approach that considers the variety of circumstances and needs of individual patients.
  - Residents, fellows and faculty members receive education on identifying and reducing health care disparities relevant to the patient population served by the clinical site.
  - Residents, fellows and faculty members receive training in cultural competency relevant to the patient population served by the clinical site.
  - Residents, fellows and faculty members know the clinical site’s priorities for addressing health care disparities.

**Slide 17:**

***HQ Pathway 6: Resident/fellow engagement in clinical site initiatives to address health care disparities^7^***

Experiential learning is essential to developing the ability to identify and institute sustainable systems-based changes to address health care disparities.

**Properties of HQ Pathway 6, include:**

Resident/fellows are engaged in QI activities addressing health care disparities for the vulnerable populations served by the clinical site.

*The focus will be on the proportion of programs that involve residents/ fellows in QI projects to reduce health care disparities, as well as on assessing whether there is some resident/fellow engagement in clinical site initiatives to address health care disparities, and resident/fellow engagement with the clinical site in defining priorities and strategies to address health care disparities specific to the site’s patient population.*

**Slide 18:**

So far, we have reviewed terms and given you an appreciation of how community service has been a part of your educational journey, as well as for your peers.

Now, we will introduce you to frameworks that can help you in transforming your service work to community service scholarship.

Given time constraints we are unable to describe in detail each framework but we would like to raise your awareness of different frameworks to consider. A framework is a model that acts as a scaffold for your research or project. It provides a structure to guide your steps and anchors the research in existing knowledge.

Ideally you should choose a framework, or frameworks, prospectively to inform your project and research. Beginning with the end in mind can assist you in ensuring that your methods and tools will be effective and valid when your research is complete. However, it is also possible to select a framework retrospectively in order to bolster your research and provide scholarly basis for your work.

**Slide 19:**

This chart provides a community engaged research framework. It outlines the importance of community assessment, problem identification, research methods, data collection, data interpretation, dissemination, and determination of impacts.^8^

The more thought you give to each, especially in considering community engagement, the better the chances are of completing community service scholarship. You do not have to use every element of a chosen framework, but the framework should fit your project’s goals and objectives.

**Slide 20:**

When developing a community service activity that involves teaching one possible framework to consider is the Kern Model.^9^ The Kern Model is a six-step approach to curriculum development which entails 1) problem identification, 2) a targeted needs assessment, 3) developing goals and objectives, 4) educational strategies., 5) implementation, and 6) evaluation and feedback.

**Slide 21:**

When developing objectives for a community service project the SMART model can be considered.

Objectives should be specific/significant, measurable,/meaningful, achieve-able/action-oriented, realistic/relevant/results-oriented, time-based/time bound/trackable.

By applying the SMART model you can create objectives for a project with greater potential for scholarship.^10^

**Slide 22:**

The Cene model is another approach to help you in transforming your service work to scholarship. This model asks you to keep in mind three important project considerations. First, the team should decide on the primary purpose of the activity. This might include education, training, clinical or community service, advocacy, policy or community-based outreach or research. Please keep in mind that many activities fulfill more than one purpose. Second, the project manager should determine the need for individual versus group involvement in the activity. Some projects can be implemented by one person, however, some require a group of individuals to carry out all tasks. Third, it is important to understand the frequency of conducting the activity to achieve the desired community-based outcome or impact of interest.^11^

**Slide 23:**

Transition slide – Let’s Practice: Sample Cases

**Slide 24-26:**

Have students break out into groups on 5-7. Distribute Case 1-3 randomly to the groups. Provide 10 minutes for the students to discuss answers to the two questions at the bottom of each case.

Ask one person per group to provide responses to the cases.

***For all three cases, trainees should comment on applying one of more of the aforementioned frameworks and models (e.g. value-based alignment, Kern Model, Cene Model, SMART, Community-Engaged Model) to their service project in order to achieve community service scholarship.***

Slide 24 Case 1(Monique): Possible models –Community-Engaged Model or Cene Model. Allow discussion of application of various models. Focus on the process Monique should go through to identify the problem, identify interventions that the student body can realistically deliver, and identify outcomes that are measurable in the short- or long-term.

Slide 25 Case 2 (Alejandro): Possible models: value-based alignment, Kern Model, Cene Model. Allow discussion of various models; focus on which elements Alejandro could change to effectuate different outcomes. Identify points of intervention and if Alejandro will create scholarly work around the curriculum transformation itself using a framework to review and adapt it, or whether he will focus on medical student learning outcomes tied to the curricular elements of change.

Slide 26 Case 3 (Jessica): Possible models: Cene model, Community-Engaged Model, value-based alignment. Allow discussion of various models. Focus on models that tie clinical or behavioral models to effecting change. Jessica could choose a framework that allows her to focus on sexual assault as a larger issue, or LGBT health and risk factors. She should also rely on ACGME competencies that relate to health disparities to justify and bolster her work (as discussed in previous slides). She could also access public health data or public safety data and tie these elements to interventions that could possibly be integrated into the ER. Jessica might also focus her project around education and intervention with the ER team itself, rather than measuring patient outcomes. She could frame her project any number of ways with different goals.

**Slide 27**

These three references are provided to illustrate examples of publications related to the cases discussed. These examples are meant to help students realize that the projects they are currently working on can have scholarship value.^12-14^

**Slide 28**

This slide provides an opportunity for a facilitator to share how they took a community service activity and transformed it into community service scholarship.^15-17^

Photos are the property JP Sanchez, MD

**Slide 29**

Research shows that roughly one-third of medical schools offer student and faculty awards for community service.^18^

Roughly 1 in 4 medical schools consider community service and engagement as part of promotion and tenure criteria.

If you are passionate about community service and engagement, it may be in your best interest to find an institution that values it in the promotion process – they exist!

**Slide 30**

Here is an example of a service portfolio from Emory School of Medicine.^19^ Notice that it includes – community activities, service related awards, advisory boards, leadership positions, editorial boards – activities for different communities.

Click next to have the smaller box appear. Remind students that in developing their service portfolio it is important to read the specific instructions listed by their home institution to ensure proper completion.

**Slide 31**

Review key points of the workshop.

**Slide 32**

Time permitting have the students reflect back on their worksheet and have them answer how their **service activity can** help build their **academic portfolio?**

**Slide 33**

Time permitting field questions. You can also have participants share their community services projects.

**References**

1. Sabo S, de Zapien J, Teufel-Shone N, Rosales C, Bergsma L, Taren D. Service learning: a vehicle for building health equity and eliminating health disparities. *American journal of public health.* 2015;105 Suppl 1:S38-43.

2. MacFarlane B. Defining and Rewarding Academic Citizenship: The implications for university promotions policy. *Journal of Higher Education Policy and Management.* 2007;29(3):261-273.

3. AAMC. *Post-MCAT Questionnaire 2015 Report.* Association of American Medical Colleges;2016.

4. AAMC. *Medical School Graduation Questionnaire 2015 All Schools Summary Report.* Association of American Medical Colleges 2015.

5. AAMC. *Medical School Graduation Questionnaire : 2014 All Schools Summary Report.* Association of American Medical Colleges 2014.

6. LCME. *FUNCTIONS AND STRUCTURE OF A MEDICAL SCHOOL, Standards for Accreditation of Medical Education Programs Leading to the MD Degree.* Liaison Committee on Medical Education 2014.

7. ACGME. *CLER Pathways to Excellence: Expectations for an optimal clinical learning environment to achieve safe and high quality patient care.* Accreditation Council for Graduate Medical Education;2014.

8. Dereski M. Collaborative Community Health Research *MedEdPortal Publications.* 2015;11.

9. Kern DE, Thomas P, Hughes M, Chen B. *Curriculum development for medical education: a six-step approach.* 3rd ed: Johns Hopkins University Press; 2015.

10. Buckner AV, Ndjakani YD, Banks B, Blumenthal DS. Using service-learning to teach community health: the Morehouse School of Medicine Community Health Course. *Academic medicine : journal of the Association of American Medical Colleges.* 2010;85(10):1645-1651.

11. Cene CW, Peek ME, Jacobs E, Horowitz CR. Community-based teaching about health disparities: combining education, scholarship, and community service. *Journal of general internal medicine.* 2010;25 Suppl 2:S130-135.

12. Allen JD, Perez JE, Tom L, Leyva B, Diaz D, Idali Torres M. A pilot test of a church-based intervention to promote multiple cancer-screening behaviors among Latinas. *Journal of cancer education : the official journal of the American Association for Cancer Education.* 2014;29(1):136-143.

13. Gonzalez CM, Fox AD, Marantz PR. The Evolution of an Elective in Health Disparities and Advocacy: Description of Instructional Strategies and Program Evaluation. *Academic medicine : journal of the Association of American Medical Colleges.* 2015;90(12):1636-1640.

14. Liebschutz J, Schwartz S, Hoyte J, et al. A chasm between injury and care: experiences of black male victims of violence. *The Journal of trauma.* 2010;69(6):1372-1378.

15. Sanchez JP, Lowe C, Freeman M, Burton W, Sanchez NF, Beil R. A Syphilis Control Intervention Targeting Black and Hispanic Men Who Have Sex with Men. *Journal of health care for the poor and underserved.* 2009;20(1):194-209.

16. Sanchez JP, Kaltwassar S, McClellan M, Burton WB, Blank A, Calderon Y. Educational video tool to increase syphilis knowledge among black and Hispanic male patients. *Journal of health care for the poor and underserved.* 2010;21(1):371-385.

17. Sanchez JP, Guilliames C, Sanchez NF, Calderon Y, Burton WB. Video tool to promote knowledge of syphilis among black and Hispanic men recruited from clinical and non-clinical settings. *Journal of community health.* 2010;35(3):220-228.

18. Goldstein AO, Bearman RS. Community engagement in US and Canadian medical schools. *Advances in medical education and practice.* 2011;2:43-49.

19. Emory University. Faculty Affairs Development Service Portfolio <http://www.med.emory.edu/administration/faculty_affairs_dev/documents/service-portfolio.html>.
