# Supplementary material for: Helping Trainees Develop Scholarship in Academic Medicine From Community Service
Source: MedEdPORTAL. 2017 Dec 14;13:10659. doi: 10.15766/mep_2374-8265.10659 (PMC6338162; doi:10.15766/mep_2374-8265.10659)
Supplement: Supplementary file 1 — A. PowerPoint Presentation.pptx B. Slide Instructions.docx C. Train the Trainer Workshop Video.mp4 D. Worksheet.doc E. Case Scenarios.doc F. Evaluation Form.doc [file mep-13-10659-s001.zip › F. Evaluation Form.doc]

**BNGAP COMMUNITY SERVICE SCHOLARSHIP EVALUATION FORM**

**Pre-Workshop Questions**

| 1. **How much CONFIDENCE do you have in your ability to…** | **No Confidence**  **0** | **1** | **2** | **3** | **Complete**  **Confidence**  **4** |
| --- | --- | --- | --- | --- | --- |
| Publish your **service**-related work | 0 | 1 | 2 | 3 | 4 |
| 2. **To what extent do you AGREE with the following statement** | **Strongly Disagree** | **Disagree** | **Neither Agree or Disagree** | **Agree** | **Strongly Agree** |
| Engaging in community-based work **does not** align with an academic medicine career | SD | D | N | A | SA |
| A career in academic medicine **would isolate** me from my **community** | SD | D | N | A | SA |
| 3. **A career in academic medicine WOULD**… | **Strongly Disagree** | **Disagree** | **Neither Agree or Disagree** | **Agree** | **Strongly Agree** |
| allow me to do work that gives me a feeling I’m serving **my community** | SD | D | N | A | SA |
| allow me to engage in **service work** in my community of interest | SD | D | N | A | SA |

**BNGAP COMMUNITY SERVICE SCHOLARSHIP EVALUATION FORM**

**Post-Workshop Questions**

| **Workshop Title and Objectives** | **To what extent do you agree that the workshop learning objectives were met** | | | | |
| --- | --- | --- | --- | --- | --- |
| **Community Service Scholarship** | **Strongly agree** | **Agree** | **Neither agree or disagree** | **Disagree** | **Strongly disagree** |
| **Obj 1: Define the terms community service, community engagement, and service learning** | SA | A | N | D | SD |
| **Obj 2: Describe frameworks to help achieve community service scholarship** | SA | A | N | D | SD |
| **Obj 3: Apply steps to achieving community service scholarship: sample cases and personal stories** | SA | A | N | D | SD |
| **What did you like about this workshop?**  **What suggestions do you have to improve this workshop?** | | | | | |
