# Supplementary material for: Helping Trainees Develop Scholarship in Academic Medicine From Community Service
Source: MedEdPORTAL. 2017 Dec 14;13:10659. doi: 10.15766/mep_2374-8265.10659 (PMC6338162; doi:10.15766/mep_2374-8265.10659)
Supplement: Supplementary file 1 — A. PowerPoint Presentation.pptx B. Slide Instructions.docx C. Train the Trainer Workshop Video.mp4 D. Worksheet.doc E. Case Scenarios.doc F. Evaluation Form.doc [file mep-13-10659-s001.zip › D. Worksheet.doc]

**COMMUNITY SERVICE SCHOLARSHIP WORKSHEET: YOUR PERSONAL STORY**

| **During medical school or residency, have you been engaged in a community service activity, that is personally gratifying but you are unsure if it bears professional value? Please describe activity.** | **What makes it personally gratifying?** | **Why are you unsure if it carries professional value?** |
| --- | --- | --- |
|  |  |  |

**COMMUNITY SERVICE SCHOLARSHIP WORKSHEET: YOUR PERSONAL STORY**

| **During medical school or residency, have you been engaged in a community service activity, that is personally gratifying but you are unsure if it bears professional value? Please describe activity.** | **What makes it personally gratifying?** | **Why are you unsure if it carries professional value?** | **How can my service activity**  **help to build my academic portfolio?** |
| --- | --- | --- | --- |
|  |  |  |  |
