# Supplementary material for: Helping Trainees Develop Scholarship in Academic Medicine From Community Service
Source: MedEdPORTAL. 2017 Dec 14;13:10659. doi: 10.15766/mep_2374-8265.10659 (PMC6338162; doi:10.15766/mep_2374-8265.10659)
Supplement: Supplementary file 1 — A. PowerPoint Presentation.pptx B. Slide Instructions.docx C. Train the Trainer Workshop Video.mp4 D. Worksheet.doc E. Case Scenarios.doc F. Evaluation Form.doc [file mep-13-10659-s001.zip › E. Case Scenarios.doc]

Monique is a 1^st^ year medical student who would like to organize a health fair at her Church which is near the medical school. She sits on the Board of a student organization and is petitioning the Board to take on this activity in the next two months. She states to the members “I grew up in the neighborhood and I’m a member of the Church, we need to do this health fair because the community is suffering from so many health disparities. They need our help!” At the end of the meeting she notices that only 2 members signed up to help with the planning and implementation.

- What would you have done differently?
- How can you help Monique transform this project from community service to scholarship?

Alejandro is a PGY-3 Pediatrics resident involved with teaching medical students. He is discussing clerkship feedback with the Chair of Pediatrics because several students mentioned that there was nothing about health disparities integrated into the rotation for morning reports or rounds. Alejandro tells the Chair he will take some of the cases and review them, adding references and examples of disparities. He also volunteers to bring in some members of the community that he works with at the free clinic in a predominantly Latino neighborhood.

- What would you have done differently?
- What are some thoughts on how to transform this community based activity into scholarship?

Jessica is a 2^nd^ year surgery resident and is concerned and saddened by the number of young men of color presenting with penetrating trauma to her hospital, some of which have had >1 assault in the past year. Among her colleagues there is a sentiment that this a long-standing problem for the local community and beyond the scope and skill-set of hospital staff. When she spoke with her residency director about inviting a Grand Rounds speaker to discuss the Epidemiology of Penetrating Trauma among Men of Color, she was told that it would provide little clinical merit.

- What would you have done differently?
- What are some thoughts on how to transform this community based activity into scholarship?
